# Development of Rapid and Economic In Vitro Assay and Biorelevant Ex Vivo Biofilm Inhibition Wound Model to Test the Antibacterial Efficacy of Wound Dressings

**DOI:** 10.1111/wrr.70080

**Published:** 2025-08-18

**Authors:** Kaisa Põhako‐Palu, Liis Preem, Kelli Randmäe, Marta Putrinš, Külli Kingo, Tanel Tenson, Karin Kogermann

**Affiliations:** ^1^ Institute of Pharmacy, University of Tartu Tartu Estonia; ^2^ Dermatology Clinic Tartu University Hospital Tartu Estonia; ^3^ Institute of Technology, University of Tartu Tartu Estonia

**Keywords:** biofilm, chronic wounds, electrospun materials, wound models

## Abstract

Chronic wounds are a major healthcare problem, consuming resources globally and necessitating innovative wound dressing development. All antimicrobial wound dressings must be tested for safety and antibacterial effectiveness prior to patient use. This study aimed to develop a rapid, economical in vitro assay and biorelevant ex vivo wound biofilm model on porcine skin to test the antibacterial efficacy of antimicrobial wound dressings. The methods were validated using five commercially available wound dressings and experimental electrospun (ES) wound dressing containing chloramphenicol in polycaprolactone and polyethylene oxide fibres (PCL/PEO/CAM). An in vitro assay was used to assess the growth inhibition, killing efficacy, and dressing sterility against multiple bacterial strains and inoculum sizes. Ex vivo models using porcine skin were used to evaluate biofilm inhibition with dressings on top of or inside infected wounds. The in vitro assay allowed rapid initial screening, whilst ex vivo models provided more biorelevant conditions for understanding the efficacy in wound‐mimicking environments. The assay and model are suitable for rapid evaluation of antimicrobial efficacy before animal studies and clinical trials. Using various commercially available wound dressings alongside novel dressings for validation ensures that the method is broadly applicable. The antibacterial efficacy of commercial antimicrobial wound dressings and experimental ES PCL/PEO/CAM fibre mat was confirmed. This study highlights the importance of using multiple complementary assays and models to comprehensively assess antimicrobial wound dressing materials.

**Abbreviations**
Ag: silver
BIM: biofilm inhibition model
BP: British Pharmacopoeia
CAM: chloramphenicol
CFU: colony forming unit
DACC: dialkylcarbamoyl chloride
DMEM/F‐12: Dulbecco's modified Eagle Medium/Nutrient Mixture F‐12 medium
EDTA: ethylenediaminetetraacetic acid
ES: electrospinning/electrospun
FBS: foetal bovine serum
H&E: Haematoxylin and eosin staining
HEPES: 4‐(2‐hydroxyethyl)‐1‐piperazineethanesulfonic acid
HPLC: high‐performance liquid chromatography
LB: lysogenic broth
NA: non‐applicable
OD: optical density
PBS: phosphate‐buffered saline
PCL: polycaprolactone
PEO: polyethylene oxide
PHMB: polyhexamethylene biguanide
RH: relative humidity
RT: room temperature
SEM: scanning electron microscopy
WBIM: wound biofilm inhibition model
WBIM‐I: wound biofilm inhibition model, dressing inside the wound
WBIM‐T: wound biofilm inhibition model, dressing on top of the wound

## 1 Introduction

Chronic wounds, where bacterial biofilms are present in about 80% of the cases, are a growing problem in society, burdening patients and healthcare systems, and consuming a great amount of healthcare resources around the globe [1, 2]. Systemic antibiotic treatment for chronic wounds is not always effective, and its widespread use increases the risk of multidrug‐resistant bacteria [3]. Functional wound dressings protect the wound, maintain a moist environment to promote healing, and possess antimicrobial properties to prevent or fight infection; they offer a promising alternative [4], but evaluating their efficacy remains challenging.

Recognised standard testing methods such as those issued by ISO, JIS, ASTM, or AATCC support reproducibility and comparability of results but often lack biological relevance, using overly simplistic conditions that fail to mimic real wound environments [5]. Therefore, there is a pressing need for advanced methods to evaluate the antibacterial efficacy of (novel) wound dressings, and customised methods hold a strong place alongside the standard methods.

Recent advances in in vitro and ex vivo wound models have improved our understanding of the antimicrobial properties of wound dressings. Unlike standard methods, novel in vitro systems simulate specific aspects of wound environments such as pH, moisture, nutrients, and oxygen availability [6]. In vitro models may combine bacteria and skin cells [7], use 3D bioprinted biofilms [8] and electrospun (ES) materials as a microenvironment for in vitro biofilm development [9]. However, these in vitro models often fail to accurately mimic real‐life biofilms, thereby limiting their clinical translatability [10]. Different in vitro methods vary considerably in setup, which in turn can affect the obtained results [11]. Ex vivo models on porcine and human skin explants closely approximate the human wound environment using natural biological tissues [12]. Porcine skin models are cost‐effective, accessible, and similar to human skin, and support biofilm growth [12, 13]. These models can be used to test the antibacterial efficacy of wound dressings in various wound types, such as burn [9, 14, 15] or biopsy wounds [16, 17], whilst full‐thickness models allow realistic biofilm studies and antimicrobial penetration assessment [18]. Biofilm models most commonly employ *P. aeruginosa* or *S. aureus* as test organisms, with a typical inoculum size of 10^5^–10^6^ CFU [19, 20]. However, other bacterial species have also been used, including *Acinetobacter baumannii* [21], *Enterococcus faecium* , and various clinical isolates [14], depending on the research focus. The maturation period for biofilm formation varies widely across studies, ranging from 2 to 72 h [14, 22]. To minimise contamination, the skin is usually pretreated; however, in some models, commensal microbes are intentionally retained to preserve a more physiologically relevant environment [14]. Strategies for reducing microbial contamination include disinfection with alcohol and/or sodium hypochlorite [21, 22], in situ generation of chlorine gas [16], and gamma irradiation [23]. Biofilm detection methods vary depending on the objective of the model but commonly include staining and imaging for qualitative or quantitative analysis [19], or physical disruption of the biofilm followed by CFU counting [14, 23].

Human skin models provide ideal conditions for testing new topical treatments, raising fewer ethical concerns than animal studies and potentially reducing the number of clinical trial volunteers [13, 24]. Whilst animal testing is crucial for obtaining biorelevant results before clinical trials [25], it is time‐consuming, costly, and ethically challenging [26]. Therefore, understanding the efficacy of novel materials in in vitro and ex vivo models is essential to reduce animal testing and clinical trials and bridge the gap between laboratory research and clinical application.

The antimicrobial activity of wound dressings depends on various factors, including the technical specifications of the testing method. Quantitative assays offer precision, whereas qualitative assays enable broader screening [27]. Static or dynamic testing conditions, single bacteria versus polymicrobial detection, and factors such as pH and antimicrobial agent release kinetics can influence the outcomes [11, 28]. Additionally, dressing composition and structural characteristics affect the adherence and stability of wounds [29]. Understanding the properties and mechanism of action of wound dressings and ensuring data are contextually evaluated helps to optimise wound treatment [30]. As new antimicrobial agents emerge, diverse analyses and combined methods are often needed to improve screening accuracy and optimise dressing design for clinical use.

This study aimed to develop and validate complementary in vitro and ex vivo antimicrobial activity testing methods for assessing wound dressings. The focus was on understanding the implications of method design on the outcomes of antimicrobial activity of wound dressings with different compositions and mechanisms of action. We developed a resource‐economical and robust in vitro assay and a more biologically relevant ex vivo model for antibacterial efficacy testing. The evaluation of the assay and model involved the use of five widely available commercial wound dressings that are commonly applied in the management of chronic and infected wounds. Furthermore, the assessment incorporated a previously developed and characterised experimental ES fibre mat loaded with the antibiotic chloramphenicol (CAM), further named PCL/PEO/CAM [31, 32].

## 2 Materials and Methods

### 2.1 Materials

#### 2.1.1 Antimicrobial Wound Dressings Used for the Validation of the Assays and Models

Altogether, 6 different antimicrobial wound dressings, including 5 commercial and 1 experimental dressing, were used to validate the in vitro assays and the ex vivo wound biofilm models (Table 1). Selected commercial antimicrobial wound dressings were AtraumanAg (Hartmann, Germany), AquacelAg + extra (Convatec, United Kingdom), Bactigras (Smith & Nephew, United Kingdom), SorbactCompress (Abigo Medical, Sweden) and SuprasorbX + PHMB (polyhexanide) (Lohmann & Rauscher, Germany). All the commercial antimicrobial wound dressings were purchased from local traders or pharmacies. For the ease of reference, the abbreviated names of the dressings are used in the text and graphs (Table 1).

**TABLE 1:** Antimicrobial wound dressings used to validate the in vitro and ex vivo wound infection models, their formulation compositions and recommended use.

Abbreviation used in the text	Brand name or explanation	Antimicrobial agent	Formulation composition	Recommended use	References
Aquacel	Aquacel Ag + extra	1.2% ionic silver, 0.39% EDTA sodium salt and benzethonium chloride	Sodium carboxymethyl cellulose dressing (combination of Hydrofiber and MORE THAN SILVER technologies)	Chronic wounds, such as (diabetic) leg ulcers and decubitus; acute burns up to 2nd degree burns.	[33, 34]
Atrauman	Atrauman Ag	Elemental silver	Polyamide fibre fabric with triglyceride‐based impregnation (caprylic/capric/myristic/stearic triglyceride; bisdiglycerylpolyacyladipate‐2 and Macrogol 2000)	Different ulcers, injuries, surgical wounds, traumatic wounds, malignant wounds and partial thickness burn wounds.	[35, 36]
Bactigras	Bactigras	0.5% chlorhexidine acetate BP	Gauze of leno weave impregnated with soft white paraffin BP	Minor burns and scalds, lacerations, abrasions and other skin loss wounds and recipient graft sites.	[37, 38]
Sorbact	Sorbact compress	NA	Dialkylcarbamoyl chloride (DACC) coated wound dressing that binds bacteria and fungi	Clean, contaminated or infected exuding wounds, such as surgical wounds, traumatic wounds, different ulcers. Both superficial and deep wounds. For managing exudate, should be used together with a secondary dressing.	[39, 40]
Suprasorb	SuprasorbX + PHMB	0.3% PHMB	Soft agar‐like dressing made up of biosynthetic HydroBalance fibres composed of fine cellulose fibrils	Superficial or deep wounds with slight to moderate amounts of exudate that are infected or at risk of infection. Can be used for all types of wound bed tissue.	[41, 42]
PCL/PEO/CAM	PCL/PEO/CAM ES fibre mat	4% CAM (w/w)	PCL 10% and PEO 2% (w/V) dissolved in a chloroform–methanol 3:1 (V/V) mixture and ES into fibre mats	NA	[31, 32]

*Note:* Information on commercial dressings was obtained from the official websites of the manufacturers and research publications.

Abbreviations: Ag, silver; BP, British Pharmacopoeia; CAM, chloramphenicol; DACC, dialkylcarbamoyl chloride; EDTA, ethylenediaminetetraacetic acid; ES, electrospun; NA, non‐applicable; PCL, polycaprolactone; PEO, polyethylene oxide; PHMB, polyhexamethylene biguanide.

The experimental ES antimicrobial wound dressing was a 4% w/w chloramphenicol (CAM)‐loaded polycaprolactone (PCL) and polyethylene oxide (PEO) fibre mat (PCL/PEO/CAM), and pristine PCL/PEO fibre mat was used as a control. Detailed information about the solution preparation and ES has been published previously [31]. The ES fibre mats were stored at a room temperature (RT) of 22°C ± 1°C and a relative humidity (RH) of 20% ± 2% until use. For quality control, the fibre mat morphology was verified using scanning electron microscopy (SEM), confirming previously reported results [31, 32, 43, 44], and the drug content was verified using high‐performance liquid chromatography (HPLC) [31, 44]. ES fibre mats were not additionally treated to eliminate potential leftover solvents from ES, as it has been previously reported that no cytotoxic effect on eukaryotic cells occurs [32, 43]. ES fibre mats were also not sterilised prior to *the* in vitro and ex vivo experiments.

#### 2.1.2 Bacterial Strains and Growth Media

Gram‐negative *Escherichia coli* ( *E. coli* ) DSM 1103 (ATCC 25922) and *Pseudomonas aeruginosa* ( *P. aeruginosa* ) DSM 1117 (ATCC 27583), and gram‐positive *Staphylococcus aureus (S. aureus)* DSM 2569 (ATCC 29213) were purchased from the Leibniz Institute DSMZ‐German Collection of Microorganisms and Cell Cultures (Braunschweig, Germany) and stored at −80°C in glycerol stocks. Bacteria were grown in BD Difco Lennox lysogenic broth (LB) (Becton, Dickinson and Company, France). For the antibacterial activity studies, Dulbecco's modified Eagle Medium/Nutrient Mixture F‐12 (DMEM/F‐12) (Sigma‐Aldrich, United Kingdom), with 100 mM 4‐(2‐hydroxyethyl)‐1‐piperazineethanesulfonic acid (HEPES) (Sigma‐Aldrich, United Kingdom) buffer, without l‐glutamine and phenol red, was used together with 10% (V/V) heat‐inactivated foetal bovine serum (FBS) (Sigma‐Aldrich, Brazil). BD Bacto Agar (Becton, Dickinson and Company, France) was used to prepare agar slurries.

#### 2.1.3 Porcine Cheek Skin Collection, Preparation and Storage

Porcine cheek skin from a local slaughterhouse (Rotaks‐R OÜ, Estonia) was used to develop an ex vivo wound biofilm inhibition model. The skin was heat‐treated in a hot water bath to remove the hair, resulting in the loss of the epidermis layer and development of superficial burn wound [9]. Porcine cheek skin pieces (approximately 10 × 10 cm) were collected manually using surgical scalpels. Samples were placed in ziplock bags, snap frozen in liquid nitrogen, and stored at −80°C. Frozen porcine skin samples on dry ice pellets were *γ*‐sterilised at 50 kGy by Scandinavian Clinics Estonia OÜ. *γ*‐Sterilisation was chosen as the least invasive sterilisation method that causes no damage to skin integrity [9]. Sterilised samples were stored at −80°C.

### 2.2 Methods

#### 2.2.1 Preparation of Overnight Cultures

Bacteria were thawed and cultured overnight on LB agar plates at 37°C. One bacterial colony from the plate was inoculated into 5 mL (in vitro assay) or 3 mL (ex vivo biofilm model) of liquid LB broth and incubated at 37°C for 18–24 h with shaking at 200 rpm. The bacterial concentration in the overnight culture was estimated based on optical density (OD) and confirmed by counting colonies of colony‐forming units (CFU) plated at 10‐fold dilutions. The 1× phosphate‐buffered saline (PBS) was used for bacterial dilution.

#### 2.2.2 In Vitro Assay in Artificial Wound Exudate

**FIGURE 1:**
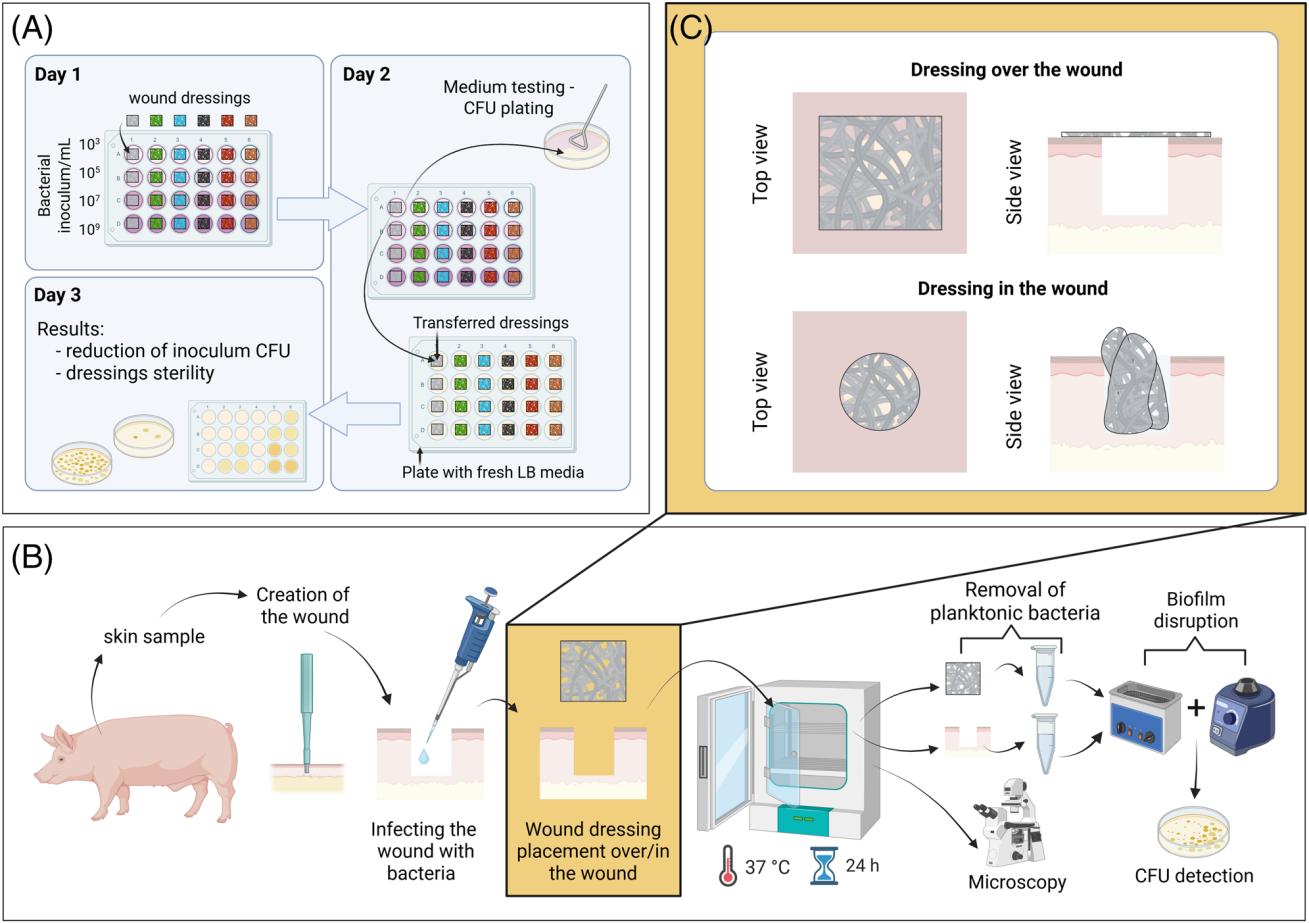
The schematic of the antibacterial activity testing using in vitro assay (a) and ex vivo wound biofilm model on porcine skin (b), where in one experiment dressings were placed on the wound and in the other part in the wound (c). Key: CFU—colony forming unit; LB—lysogenic broth.

Overnight liquid cultures of *E. coli* DSM1103, *S. aureus* DSM2569 and *P. aeruginosa* DSM1117 were centrifuged at 3000 rpm for 10 min. The supernatants were removed, and the pellets were resuspended in artificial wound exudate composed of DMEM/F‐12 without phenol red and supplemented with FBS (10% (V/V)). Each culture was diluted to an OD of 1 at 600 nm, resulting in approximately 10^9^ CFU/mL. Four inoculum sizes were prepared, with serial dilutions of 10^9^, 10^7^, 10^5^, and 10^3^ CFU/mL. The 1 cm^2^ pieces of tested dressings were transferred into individual wells of a 24‐well plate and immersed in 1 mL of a single bacterial dispersion (Figure 1a). After 24 h of incubation at 37°C, the turbidity of the medium in each well was checked to evaluate the dressing growth inhibition potential. This was not possible in wells with an initial 10^9^ CFU/mL due to high culture density and resulting turbidity, even if all bacteria were killed. To measure viable bacteria in the growth medium and inoculum CFU reduction, 100 μL of the medium from each well was plated on LB agar plates and incubated at 37°C until colony counting was performed the next day. To check for viable bacteria attached to dressings, they were washed twice with 1× PBS to remove medium residues and loose bacteria, then transferred into 1 mL of fresh LB liquid medium on a new 24‐well plate. The plate was incubated at 37°C until the following day, when sterility was estimated. Medium turbidity indicated viable bacteria attached to the dressing, whereas clear medium indicated sterility.

#### 2.2.3 ASTM E2180‐18 In Vitro Assay

The method followed the ASTM E2180‐18 standard with minor modifications. A 0.3% agar slurry was prepared by heating and stirring agar in 1× PBS until fully dissolved, then sterilised by autoclaving for 15 min at 121°C and equilibrated to 45°C.

Overnight liquid cultures of *E. coli* DSM1103, *S. aureus* DSM2569, and *P. aeruginosa* DSM1117 were diluted to OD (600 nm) of 1, 0.1, and 0.0001. A 100 μL aliquot of each bacterial dispersion was added to a separate 10 mL portion of agar slurry, creating slurries with three different inoculum sizes. CFU counts were determined by dilution and plating.

A 1 cm^2^ piece of Atrauman and Sorbact hydrophobic dressings was placed in individual wells of a 24‐well plate, and 100 μL of inoculated agar slurry was pipetted onto each piece. As a control, 100 μL of the inoculated agar slurry was pipetted into empty wells. To prevent drying, 1 mL of 1× PBS was added to the outer empty wells, and the plates were sealed in plastic ziplock bags. Once the slurry had gelled, the plates were incubated at 37°C for 24 h. Three replications were performed for each dressing and control.

After incubation, the dressings were transferred into empty Eppendorf tubes. The wells from which dressings were removed were rinsed with 1 mL of 1× PBS, and rinsate was added to the respective tubes containing dressings. Control wells were similarly rinsed with 1 mL of 1× PBS, and the rinsates were transferred into empty Eppendorf tubes.

The tubes were then sonicated for 1 min and vortexed at the maximum speed for 1 min. Ten‐fold serial dilutions were prepared in 1× PBS and plated on LB plates for bacterial quantification.

#### 2.2.4 Ex Vivo Wound Biofilm Inhibition Models on Porcine Skin

An ex vivo wound biofilm inhibition model on porcine skin was developed, based on a previously published biofilm model [9]. The experimental setup was as follows (Table 2):

1. BIM‐T—heat‐treated infected porcine skin without biopsy wound;
2. WBIM‐T—heat‐treated porcine skin with infected biopsy wound and treatment dressing on top of the wound; and
3. WBIM‐I—heat‐treated porcine skin with infected biopsy wound and treatment dressing inside the wound.

**TABLE 2:** Description of models used in ex vivo wound biofilm model development.

Model	Heat treatment	4 mm biopsy wound	*E. coli* infection	Treatment with dressing
BIM‐T	+	−	+	On top
WBIM‐T	+	+	+	On top
WBIM‐I	+	+	+	Inside

*Note:* Key: + (red)‐ the feature was present in the model; ‐ (white) ‐ the feature was not present in the model.

Abbreviations: BIM, biofilm inhibition model; I, inside; T, on top; WBIM, wound biofilm inhibition model.

Negative control without infection and treatment validated porcine skin sterility; positive control with infection and without treatment validated biofilm formation.

The ex vivo WBIM models (Figure 1b) were constructed in sterile flat‐bottom 24‐well plates (VWR International LLC, China). DMEM/F‐12 medium without l‐glutamine and phenol red, supplemented with heat‐inactivated FBS (10% (V/V)) mimicked wound exudate and kept the skin moist. The 250 μL medium was added to each well. Thawed heat‐treated porcine cheek skin was cut into 1 × 1 cm pieces and wounded using a 4 mm biopsy needle (KAI Medical, Japan). Skin samples were placed into the wells and moisturised with sterile 1× PBS. The wound was infected with 5 μL of bacterial suspension, made from an overnight culture (LB broth, 20 h, 37°C, 200 rpm) diluted to 10^6^ CFU/mL using 1× PBS. To assess the antibacterial activity and biofilm inhibition, commercial wound dressings and ES fibre mats were placed over or in the wound (Figure 1c). In a separate experiment, Aquacel and Bactigras dressings were moisturised using 100, 200, and 300 μL of DMEM/F‐12 medium for Aquacel and 25, 50, and 75 μL for Bactigras. Well plates were sealed with parafilm, placed in ziplock bags to maintain moisture, and incubated at 37°C for 24 h.

After incubation, bacterial growth was measured by counting the CFUs. To remove planktonic bacteria, wound dressings or ES fibre mats were removed from the skin and put into a 1.5 mL Eppendorf tube with 1 mL of 1× PBS. The same procedure was performed for the skin. The tubes were vortexed at maximum speed for 3 s. Wound dressings or ES fibre mats and skin samples were placed into a new tube with 1 mL of 1× PBS, and the biofilm was disrupted. For that, samples were vortexed at maximum speed for 30 s, followed by sonification for 30 s. This procedure was repeated six times. The samples were kept on ice when not handled. After biofilm disruption, 100 μL aliquots from samples in 1× PBS were used to make 10‐times dilutions, plated on LB agar plates, and incubated overnight at 37°C. Rinsed‐off planktonic bacteria in 1× PBS were also plated and incubated similarly (results in Supporting Information). All experiments were performed in triplicates, with three technical replicates for CFU counting.

#### 2.2.5 Paraffin‐Embedding of Porcine Skin Samples for Microscopy Imaging

Ex vivo wounded and infected porcine skin samples treated with ES fibre mats PCL/PEO (control mat) and PCL/PEO/CAM and control samples with and without infection were created as described in the previous section. After 24 h of incubation at 37°C, skin samples with ES fibre mats were placed in 10% (V/V) formaldehyde in 1× PBS solution. For sample dehydration and cleaning, a vacuum infiltration processor Tissue‐Tek VIP 5 Jr. (Sakura, USA) was used. The skin samples were then placed in paraffin using the Paraffin Embedding Centre EG 1160 (Leica, Germany) and stored at RT.

#### 2.2.6 Haematoxylin and Eosin (H&E) Staining, Visualising and SEM Imaging of Histological Samples

Paraffin‐embedded samples were cut into 6 μm slices using a microtome (Microm HM 360). Slices were placed in a warm water bath and caught on microscope glass slides (SuperfrostTMPlus Adhesion Microscope Slides, Epredia, USA) and left at 37°C overnight to seal. Slides were de‐paraffinised in xylene for 4 min, then rehydrated: 2 min in absolute ethanol (WVR, USA), 2 min in 95% ethanol, 2 min in 75% ethanol, and 5 min in tap water. The samples were placed in haematoxylin solution (Sigma‐Aldrich, USA) for 10 min and washed with tap water. For eosin staining, the slides were placed in eosin solution (Histolab, Sweden) for 10 s and washed in tap water. The slides were left in tap water for 5 min, then dehydrated: 1 min in 70% ethanol, 1 min in 95% ethanol, and 1 min in absolute ethanol. Slides were cleared in xylene for 1 min, mounted with a cover glass and Pertex mounting medium (Histolab, Sweden). Imaging was performed using a Pannoramic 250 Flash III digital scanner (3DHistech Ltd., Hungary). Images were processed using the SlideViewer software.

Additionally, SEM was used to visually evaluate the antibiofilm effect of the ES fibre mats in an ex vivo wound biofilm inhibition model. Before SEM imaging, paraffin‐embedded skin samples were prepared and de‐paraffinised as described in the previous paragraph. De‐paraffinised slides were air‐dried and cut into a suitable size using a diamond knife to be placed on the SEM sample stubs. The samples were then coated with 5 nm platinum using a Q150T‐ES Sputter Coater (Quorum, United Kingdom). The samples were imaged using a Merlin FE‐SEM microscope (Carl Zeiss, Germany).

#### 2.2.7 Data Analysis

The ES fibre size distribution was manually measured from two different SEM images using the ImageJ software. The number of measured fibres was *N* = 115 for the PCL/PEO ES fibres and *N* = 195 for the PCL/PEO/CAM ES fibres.

The log reduction in bacterial growth after treatment with the different dressings was calculated using the following formula:

$$\mathrm{Log}\;\text{reduction}=\log_{10}\left(A/B\right)$$

where *A* is the number of bacteria in the initial inoculum and *B* is the corresponding number of bacteria detected after the dressing treatment.

The CFU data were log‐transformed prior to statistical analysis. The results are expressed as the mean of at least three biological and three technical replicates ± standard deviation (SD). Statistical analysis was performed using one‐way ANOVA and post hoc Tukey's multiple comparisons test using GraphPad Prism 5 software (*p* < 0.05). GraphPad Prism 5 and BioRender.com were used to create the graphs and figures, respectively.

## 3 Results

### 3.1 Characterisation of Commercially Available Antimicrobial Wound Dressings and Electrospun Fibre Mats

All tested commercial antimicrobial wound dressings were selected based on the clinical indications for infected wound treatment and their composition (Table 1). Experimental ES fibre mats containing CAM were selected due to previously reported antibacterial effects on relevant wound pathogens [9, 31, 32].

**FIGURE 2:**
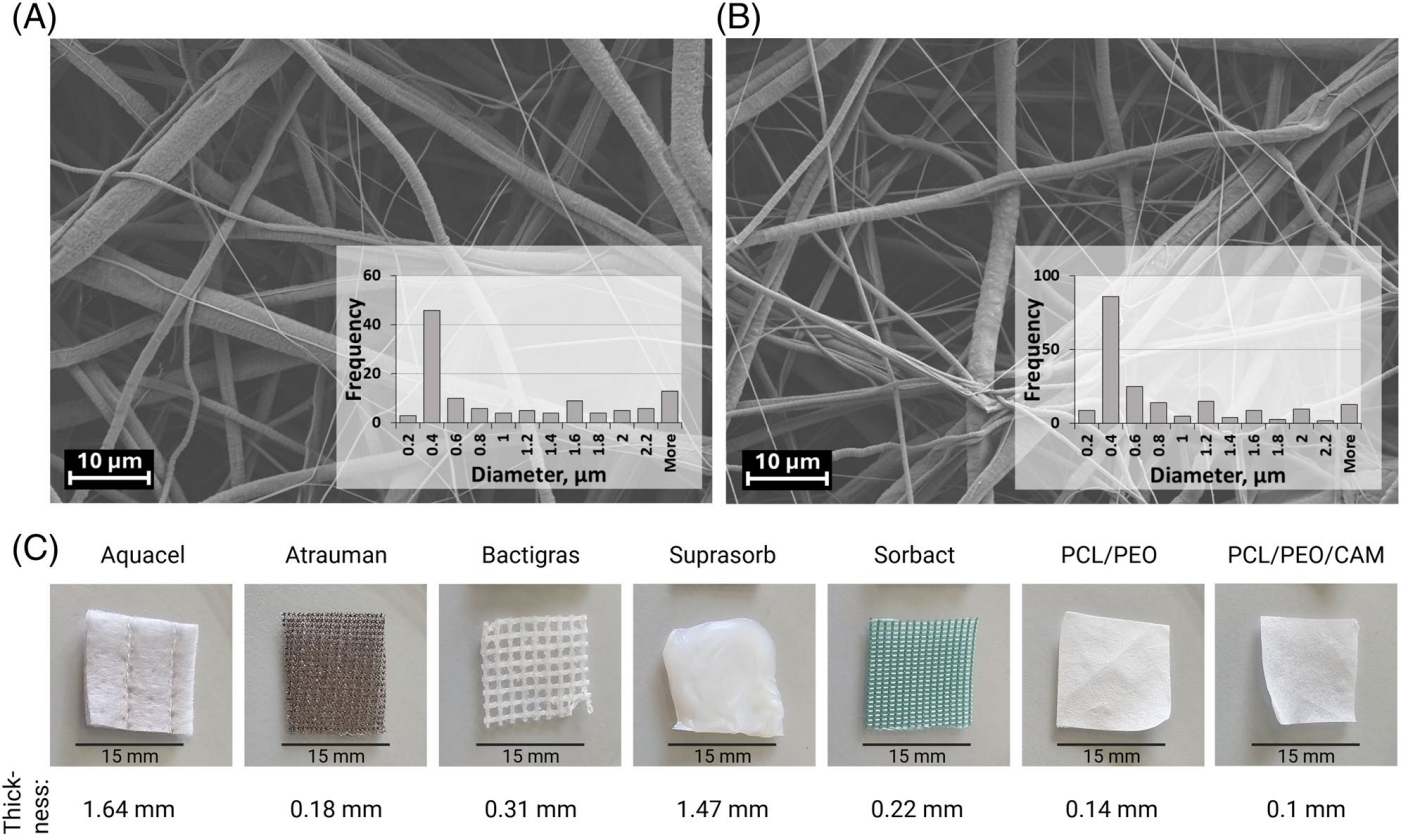
Scanning electron microscopy (SEM) images together with fibre diameter size distribution histograms (*N* = 115–195 fibres) of ES PCL/PEO fibre mat (a) and PCL/PEO/CAM fibre mat (b). Photographs of all wound dressings used in this study (c). Key: CAM—chloramphenicol; PCL—polycaprolactone; PEO—polyethylene oxide.

All dressings have fibrous structures, but differ in fibre size and orientation, hydrophilicity or hydrophobicity, water content, thickness, antimicrobial agents, and antimicrobial mechanisms (Table 1 and Figure 2). Figure 2c shows the visual representations and thickness values for each dressing. Aquacel, Suprasorb, and ES fibre mats have nonwoven structures, whilst Atrauman, Bactigras, and Sorbact are woven fabrics. Suprasorb is a soft hydrogel, Aquacel is cotton‐like, and both are thicker than other dressings. Atrauman and Bactigras have grid‐like structures impregnated with neutral lipids or white soft paraffin, respectively, making them hydrophobic. Aquacel, Suprasorb, and PCL/PEO/CAM ES fibre mat are hydrophilic. ES fibre mats have a smooth white surface resembling thin tissue paper with nano‐ and microfibrous structure visible under SEM. Fibre diameters vary throughout the mats (Figure 2a,b), ranging from 0.2 to 5 μm for PCL/PEO/CAM and pristine PCL/PEO fibre mats, matching previous results [43]. A broader distribution of fibre sizes can be beneficial in the context of wound healing, as the native extracellular matrix of the skin also comprises fibres with varying morphologies. Mimicking this structural variability has been shown to enhance biological performance [45]. To ensure consistency and quality, each batch of ES fibres was tested for quality by SEM analysis for morphology and size distribution assessment, and HPLC analysis for antimicrobial agent content. Notably, the HPLC results (data not shown) align with previous studies by Preem et al. [31, 32], reinforcing the reliability of our methodology.

### 3.2 In Vitro Antibacterial Activity Assay

**FIGURE 3:**
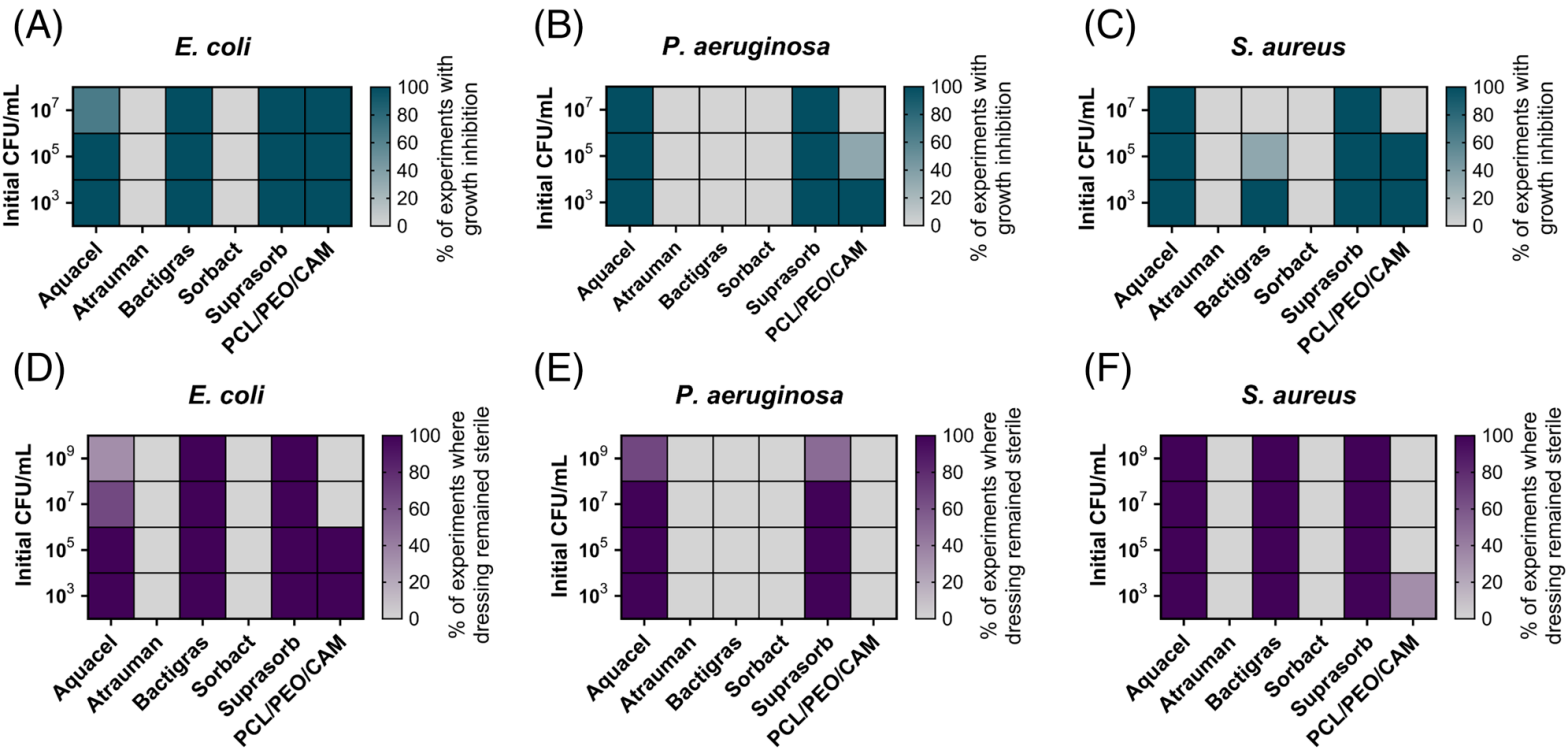
Growth inhibition of pathogens *E. coli* (a), *P. aeruginosa* (b) and *S. aureus* (c) in biorelevant medium (DMEM/Ham‐12) after 24 h. Dressing sterility after 24 h in vitro experiment with pathogens *E. coli* (d), *P. aeruginosa* (e) and *S. aureus* (f). The data are represented as a mean of three biological replicates (*N* = 3). Key: CAM—chloramphenicol; CFU—colony forming unit; PCL—polycaprolactone; PEO—polyethylene oxide.

**FIGURE 4:**
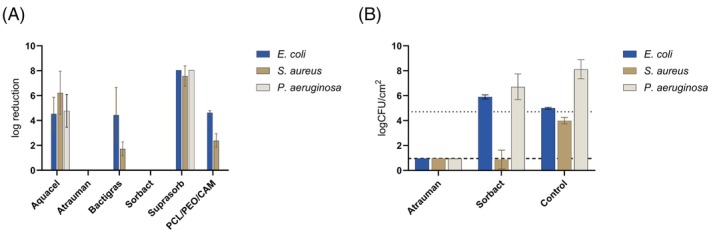
The log reduction values of different antimicrobial wound dressings in our in vitro assay with pathogens *E. coli* , *P. aeruginosa*, and *S. aureus* (a). Mean and standard deviation, SD (*N* = 3) of the log‐transformed number of *E. coli* DSM1103, *S. aureus* DSM2569 and *P. aeruginosa* DSM1117 CFUs detected on Atrauman and Sorbact dressings, and in control wells in ASTM E218‐18 assay (b). The dotted line represents the number of bacteria in the initial inocula (logCFU/cm^2^ of dressing), and the dashed line represents the detection limit. The data are represented as a mean and SD of at least three biological replicates. Key: CAM—chloramphenicol; CFU—colony forming unit; PCL—polycaprolactone; PEO—polyethylene oxide.

The in vitro method assessed how effectively each dressing inhibited bacterial growth or killed bacteria when challenged with different bacterial strains and inoculum sizes, and whether the dressing remained sterile (Figures 3 and 4a).

The test revealed that when exposed to *E. coli* , most dressings limited bacterial growth even at high inoculum levels (Figure 3a); whereas the effect against *S. aureus* was reduced (Figure 3c) and only two dressings were effective against *P. aeruginosa* (Figure 3b). These two dressings—Aquacel and Suprasorb—showed consistently high activity against all tested bacteria. Comparing killing efficacy (Figure 4a), Suprasorb demonstrated the strongest bactericidal properties, with log reduction values of 7.5 for *S. aureus* and above 8 for gram‐negative bacteria. Aquacel's killing efficacy was also good, with log reduction values from 4.5 to 6.2 (Figure 4a). These dressings also remained sterile under most conditions; although at higher inoculum levels, contamination increased. Aquacel sterility was less than 100% starting from an *E. coli* inoculum of 10^7^ CFU/mL and from a *P. aeruginosa* inoculum of 10^9^ CFU/mL (Figure 3d,e). Suprasorb's sterility was compromised only when exposed to 10^9^ CFU/mL of *P. aeruginosa* (Figure 3e).

Bactigras dressing showed good bacteriostatic and bactericidal activity against *E. coli* (Figures 3a and 4a). The dressing remained sterile at the highest inoculum level tested (Figure 3d). However, its activity was lower against *S. aureus* and absent against *P. aeruginosa* . Whilst Bactigras' activity against *S. aureus* was modest, the dressing remained sterile even at the highest inoculum level.

The ES dressing PCL/PEO/CAM exhibited good bacteriostatic and bactericidal activity against *E. coli* and limited activity against *S. aureus* (Figures 3c and 4a). PCL/PEO/CAM showed bacteriostatic activity against *P. aeruginosa* , despite CAM's inherently low activity against this pathogen [46] (Figure 3b). No killing of *P. aeruginosa* was observed (Figure 4a). The dressing remained sterile only at lower *E. coli* inoculum levels. Two commercial dressings—Sorbact and Atrauman—showed no antibacterial activity in our in vitro assay, even at the lowest inoculum sizes.

### 3.3 ASTM E2180‐18 In Vitro Assay

Since neither Atrauman nor Sorbact dressings rely on releasing and diffusing active ingredients into the bulk liquid for their effects, no antibacterial activity was observed in our developed in vitro method. These dressings were further tested using the ASTM E2180‐18 assay, a standard method for determining the activity of incorporated antimicrobial agent(s) in polymeric or hydrophobic materials. This method was chosen because both dressings are hydrophobic, and the method allows us to test their antimicrobial effect considering their expected mechanisms of action. This method has previously been used to test Atrauman dressing's antibacterial activity [35]. The method was modified by replacing saline with 1× PBS as the agar slurry base, maintaining the pH within a more physiologically relevant range.

The results showed that the Atrauman dressing was effective against all tested bacteria, producing at least 3 log reductions (Figure 4b). No bacteria were detected in these samples, with a detection limit of 10 CFU/cm^2^ dressing. The Sorbact dressing was effective against *S. aureus* but not against gram‐negative bacteria. Interestingly, the controls of different bacteria behaved differently. *E. coli* CFU‐s remained approximately the same as in the initial inoculum, whereas *S. aureus* numbers were about 10 times reduced, and *P. aeruginosa* increased approximately a 1000 times.

As we saw in our developed in vitro assay, inoculum size is an important factor in determining the efficacy of a dressing against bacteria. Hence, we tested the hypothesis that the efficacy of Sorbact dressing could be observed in the ASTM assay if the number of bacteria it is challenged with is reduced. However, this proved to be incorrect, as even when the dressing was challenged with less than 100 CFU/cm^2^, no effect was observed. By comparison, Atrauman was active even when the inocula were increased (Figure S1).

### 3.4 Ex Vivo Biofilm Wound Models on Porcine Skin

In addition to the in vitro assay, a more biorelevant ex vivo wound biofilm inhibition model (WBIM) was developed using heat‐treated biopsy‐wounded porcine cheek skin to mimic the infected wound conditions. Two WBIM setups were used, with the dressing placed on top (WBIM‐T) or inside the wound (WBIM‐I) (Figure 1c). For comparison, a biofilm inhibition model (BIM) without a biopsy wound, based on Lorenz et al. [9] work, was used. In all ex vivo model experiments, the antimicrobial efficacy of the wound dressings was tested against *E. coli* DSM1103 strain. *E. coli* was chosen as the main cause of bacterial infections in chronic wounds; Gram‐negative bacteria, with *E. coli* being the second leading cause [47].

**FIGURE 5:**
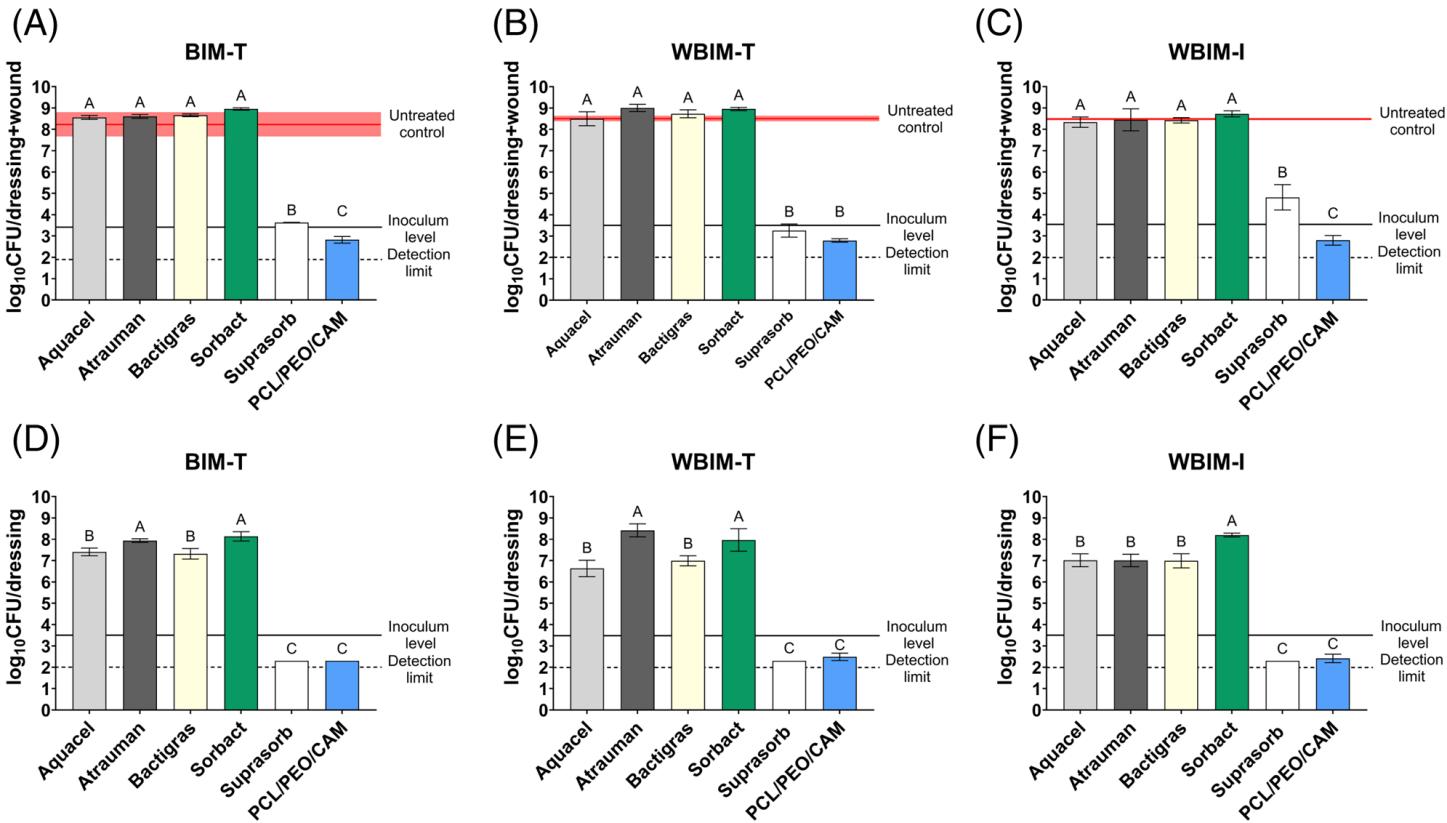
Mean of log‐transformed total (CFU/wound + dressing) *E. coli* bacterial growth on BIM (a), WBIM‐T (b) and WBIM‐I (c) and mean of log‐transformed *E. coli* bacterial growth on tested wound dressings only in BIM (d), WBIM‐T (e) and WBIM‐I (f). Red line marks mean untreated control bacterial levels with SD (pink area), solid black line indicates inoculum level of 5 × 10^3^ and dotted line indicates detection limit of 200 CFU. Data for commercial antimicrobial wound dressings are represented as the mean of three biological replicates (*N* = 3), and data for ES materials as the mean of nine biological replicates (*N* = 9). Key: BIM—biofilm inhibition model; CFU—colony forming unit; CAM—chloramphenicol; PCL—polycaprolactone; PEO—polyethylene oxide; WBIM—wound biofilm inhibition model; T—on top; I—inside. The GraphPad compact letter display feature indicates statistically significant differences between dressings in the pairwise comparisons. In each model, no statistically significant differences were observed between dressings sharing a letter.

Bacterial growth was detected separately for wound dressings and porcine skin samples, and the total bacterial numbers (CFU/wound + dressing) are shown in Figure 5. For untreated controls, the CFU/wound was approximately 10^8^ in all models (Figure 5, red line, pink area represents SD). The same total number of bacteria with no statistically significant differences was detected for Atrauman, Aquacel, Bactigras, and Sorbact dressings in all ex vivo models. The mean planktonic bacterial count was approximately 10^8^ in all models for these dressings (Figure S2).

Suprasorb and PCL/PEO/CAM fibre mat were the only dressings with antibacterial effect in ex vivo models. For Suprasorb, the total average CFU/wound + dressing was 10^3^ in BIM and WBIM‐T (Figure 5a,b), and 10^5^ in WBIM‐I (Figure 5c). For PCL/PEO/CAM, the mean total CFU/wound + dressing was near the detection limit of 200 CFUs in all ex vivo models (Figure 5, dotted line). PCL/PEO/CAM fibre mat was more effective than Suprasorb in the BIM‐T and WBIM‐I models, as they were placed in different groups (B and C) by statistical analysis.

Secondly, we examined bacterial growth on wound dressings used in ex vivo model experiments, considering their potential as surfaces for bacterial attachment and contamination sources. Atrauman, Aquacel, Bactigras, and Sorbact dressings were good substrates for biofilm formation, with variations in detected CFUs between models (Figure 5d–f). In BIM and WBIM‐I, bacterial growth on Atrauman, Aquacel, and Bactigras dressings was approximately 10^7^ CFU/dressing and on Sorbact dressing 10^8^ CFU/dressing (Figure 5d,f). WBIM‐T showed greater variation in bacterial growth on the wound dressings. Detected bacterial levels were approximately 10^8^, 10^6^, 10^7^ and 10^8^ CFU/dressing for Atrauman, Aquacel, Bactigras, and Sorbact, respectively (Figure 5e). Sorbact had the highest number of attached bacteria, explained by its mechanism to bind bacteria [39]. However, the binding should be irreversible, with no viable bacteria coming loose. Sorbact was unable to bind all loose bacteria from the wound, as bacterial growth on the skin was similar to the untreated control at 10^8^ CFU in all models (Figure S3). This applies to Atrauman, Aquacel, and Bactigras dressings as well. Suprasorb was the only dressing that remained sterile in all ex vivo models, and little bacterial growth, near the detection limit, was observed on the ES PCL/PEO/CAM fibre mat.

**FIGURE 6:**
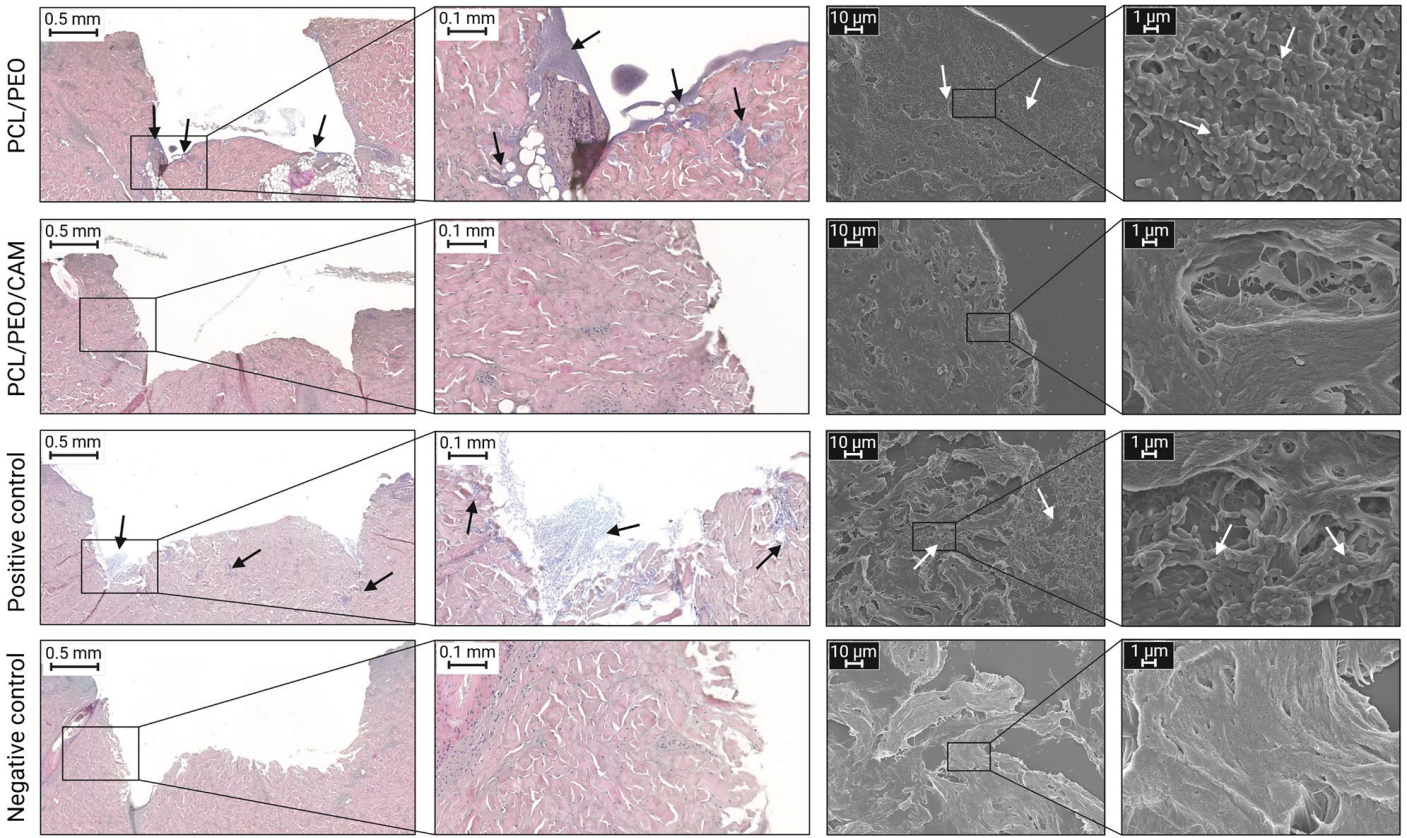
Haematoxylin and eosin (H&E)‐stained histological images together with scanning electron microscopy (SEM) images of ex vivo porcine skin samples treated with PCL/PEO and PCL/PEO/CAM ES fibre mats for 24 h. The black and white arrows mark the *E. coli* DSM1103 formed biofilm. Key: CAM—chloramphenicol; PCL—polycaprolactone; PEO—polyethylene oxide; positive control—heat‐treated, biopsy‐wounded and infected untreated porcine skin sample; negative control—heat‐treated, biopsy‐wounded and uninfected untreated porcine skin sample.

We performed histological analysis of PCL/PEO/CAM fibre mat‐treated skin samples to visualise the antibiofilm and antibacterial efficacy. Skin treated with the PCL/PEO ES fibre mat was analysed as a control for a dressing without antibacterial properties. Samples from the ex vivo WBIM‐T model were visualised using H&E staining, which identifies bacterial aggregates [48]. Bacterial aggregates are stained purple and are smaller than the surrounding skin cells [49]. SEM imaging confirmed the H&E staining results, providing detailed morphological evidence of the presence or absence of bacteria within the treated samples. Bacterial biofilm formation was observed in the untreated infected control (positive control), whereas no infection was seen in the negative control (Figure 6). In the positive control, the bacterial biofilm penetrated deep into the skin layers, highlighting the complexity and resilience of the biofilm structures in a wound‐like environment. Biofilm formation was also visible in samples treated with the ES PCL/PEO fibre mat, suggesting that the absence of antimicrobial agents in the fibre mat limits its ability to inhibit bacterial colonisation.

In contrast, no bacterial growth was observed in porcine skin samples treated with ES PCL/PEO/CAM fibre mats, indicating successful antibacterial action. This demonstrates the effective release and activity of the antimicrobial agent CAM from ES fibres, aligning with the CFU counting results. The correlation between the histological, SEM, and microbiological findings reinforces the reliability of our model for assessing the antibacterial efficacy of wound dressings. Furthermore, these results emphasise the necessity of validating novel antimicrobial dressing methods using histological and biofilm visualisation techniques in addition to standard microbiological assays. Preventing biofilm formation is critical for chronic wound management, where biofilms contribute to persistent infections and delayed healing.

Based on these results, we hypothesised that the lack of antibacterial activity in some dressings in ex vivo models could be due to insufficient moisture levels needed for drug release. Since Aquacel and Bactigras dressings showed better results against *the E. coli* DSM1103 strain in vitro, but no ex vivo efficacy, we modified the ex vivo models by adding different amounts of DMEM/Ham‐12 medium to promote the release of antimicrobial agents. The inhibitory effect of Bactigras has been shown to increase with serum or saline addition [37], and Aquacel dressing has been pre‐moistened before application in ex vivo porcine biofilm models [19]. We added 100, 200, or 300 μL of medium to the Aquacel‐treated models and 25, 50, or 75 μL to Bactigras‐treated models. The results showed that wetting the models did not significantly affect the effectiveness of Aquacel and Bactigras dressings in preventing the development of infection (Figure S4). There was also large variability in Aquacel‐treated model results, which was not observed in non‐wetted versions (Table S1).

## 4 Discussion

Special attention has been paid to developing chronic wound and wound infection models that are biorelevant and easy to use [12]. As a simplification of reality, a model does not need to be perfect to be useful; understanding what it can mimic, its advantages, disadvantages, and implementation for characterisation is the key. In this article, different in vitro antibacterial activity assays and ex vivo wound biofilm inhibition models were developed (Figure 1) and validated with both commercially available antimicrobial wound dressings with clinically proven efficacy and an experimental antibiotic‐loaded ES wound dressing (Table 1 and Figure 2). In addition to our experimental validation, we systematically reviewed previously reported models used for testing the antimicrobial efficacy of selected commercial wound dressings, as detailed in Table S2.

The developed in vitro assay was easy‐to‐use and economical, enabling the screening of multiple dressings against various bacterial strains and inoculum sizes, assessing growth inhibition, killing efficacy, and dressing sterility. Validation using different wound dressings highlighted the strengths and limitations of the assay. Whilst versatile and informative, it was unsuitable for dressings requiring direct contact with pathogens, such as those relying on binding and sequestering microbes or contact‐killing via mechanical or chemical interactions [50, 51]. Whilst other dressings released their active ingredient(s) into the liquid medium and were more or less effective against pathogens, Atrauman and Sorbact lacked the effect in our assay (Figures 3 and 4).

These dressings were additionally tested using a standard ASTM E2180‐18 assay developed for hydrophobic dressings relying on direct contact rather than antimicrobial agent release. Atrauman generates silver ions upon contact with wound exudate, becoming bactericidal upon direct contact with bacteria [35], whilst highly hydrophobic DACC‐coated Sorbact irreversibly adsorbs pathogens via hydrophobic interactions [39]. Using the ASTM E2180‐18 assay, Atrauman showed efficacy against all three tested pathogens (Figure 4b) and has previously demonstrated to be effective against several other pathogens using the same method [35], whereas Sorbact demonstrated limited effectiveness only against *S. aureus* (Figure 4b). This may be due to the sonication and vortexing methods used to detach bacteria, which can break the bonds between Sorbact's DACC coating and bacteria, releasing viable pathogens. Sorbact's efficacy against *S. aureus* and other pathogens has been shown with the JIS L1902 method using Tween 20 to aid bacterial extraction [39]. However, sonication was probably not the only factor, as bacterial numbers on the dressing sometimes increased, indicating replication despite the dressing's presence. It was challenging to uniformly wet the Sorbact dressing with agar slurry, even with added surfactants (data not shown). As a result, the contact between the dressing and bacteria may have been insufficient. The solidification of the agar likely worsened the issue, reducing bacterial contact with the dressing.

The main disadvantage of ASTM E2180‐18 is its lack of biorelevance for testing wound dressings. Whilst PBS was used for pH control, a more complex medium could be used to mimic wound exudate and support bacterial growth. Modifying the method with different media, inoculum sizes, and conditions can enhance its versatility. In our developed in vitro assay, artificial wound exudate was simulated using DMEM/F‐12 medium supplemented with FBS. The added serum interacted with bacteria and dressings, providing more biologically relevant data. Previous studies have shown that serum significantly reduces Sorbact's antimicrobial activity, likely due to bacterial agglomerates forming within fibre grates under high‐protein conditions [39], which may explain the lack of effect in our assay. A high protein content has also been shown to strongly reduce or inhibit silver's antimicrobial activity [52], highlighting the importance of using biorelevant media in antibacterial activity testing.

We tested various inoculum sizes in our in vitro assay and observed that increasing the inoculum size posed challenges for some dressings (Figure 3). Suprasorb maintained bactericidal activity at high inoculum levels, whereas other dressings showed varying efficacy depending on the inoculum size. The inoculum effect, where the activity of antibiotics [53, 54], antimicrobial peptides [55], and antimicrobials, such as silver [56], depends on the initial cell density in bacterial growth inhibition assays, questions the reliability of standardised tests using a single inoculum density. Husmark et al. [39] found that increasing inoculum densities also reduced the binding‐based antimicrobial efficacy of Sorbact in the JIS L 1902 contact test. Testing across inoculum sizes provides insights into dressing performance and suitability for varying wound contamination levels, from clean surgical wounds to heavily infected chronic wounds.

One often overlooked aspect is that wound dressings can become contamination sources as bacteria may form biofilms on their surfaces under favourable conditions [9]. The ability of dressing materials to facilitate biofilm formation can vary significantly [31]. In our in vitro assay, we observed that the dressing's ability to maintain sterility is influenced by inoculum size, similar to other antibacterial properties (Figure 3). Bactigras remained sterile even at high bacterial densities (10^9^ CFU/mL), although it effectively inhibited bacterial growth only at lower inoculum levels (10^5^–10^7^ CFU/mL). Conversely, the PCL/PEO/CAM fibre mat's ability to maintain sterility was less effective than its potential to inhibit bacterial growth. As the PCL/PEO/CAM fibre mat rapidly releases its active ingredient upon contact with the medium, bacteria can attach once the active ingredient is depleted [31]. Bactigras may retain effective drug levels for 24 h [57], preventing bacterial growth on its surfaces. In addition, factors such as surface hydrophobicity/hydrophilicity, charge, roughness, and topography can influence bacterial adhesion [58]. Because not all dressings remained sterile when challenged with certain bacterial species or concentrations, incorporating this aspect into antibacterial activity testing adds significant value to the assay.

Although in vitro assays can be made more biorelevant, mimicking the complexity of biological systems remains challenging. Ex vivo models offer a practical alternative, using native skin tissue to study antimicrobial action without costly, time‐intensive animal models. This study compared three ex vivo wound biofilm inhibition models using porcine skin: BIM, WBIM‐T, and WBIM‐I (Table 2 and Figure 1). All three models showed similar trends with minor differences in the antibacterial efficacy of the tested dressings against *E. coli* (Figure 5). This suggests that the models are essentially equivalent; despite the increased complexity of WBIM models, they do not provide additional insights compared to the BIM model. Therefore, the simplest ex vivo model can be chosen if it yields comparable outcomes, thereby avoiding unnecessary complexity.

The antimicrobial effectiveness of the tested dressings varied significantly between in vitro assays and ex vivo models. Whilst Aquacel and Bactigras dressings showed strong activity against *E. coli* in vitro, they failed to inhibit bacterial growth ex vivo (Figure 5). These differences can be attributed to several factors. A major factor is the complexity of porcine cheek skin as a substrate, which allows bacteria to migrate into deep tissue pockets, creating protective niches, as observed in histological images (Figure 6), and allowing biofilm formation. Although planktonic bacteria were used in both models, the transition from planktonic to biofilm can be more favoured on the skin. As biofilms are harder to treat [59], this could explain the difference in efficacy. Additionally, the active concentration of antimicrobials at the target site can be reduced by limited diffusion through the skin and binding to proteins [60]. Penetration of bacteria through the skin explants and the resulting difficulties in antimicrobial activity assessment has also been observed by Yang et al., who addressed this issue by placing *P. aeruginosa* PAO1‐inoculated skin explants on 0.5% TSA agar supplemented with 50 μg/mL gentamicin to restrict biofilm formation on the skin surface [16]. However, introducing antibiotics into the ex vivo system may also alter the test outcomes and reduce the biological relevance of the model.

The liquid amount in the ex vivo models differed from that in the in vitro setup, potentially influencing drug release and diffusion. To test whether the ex vivo models were too dry, we introduced varying amounts of medium to mimic wound exudate. However, the results did not support this hypothesis and showed increased intra‐assay variability (Figure S3 and Table S1). This illustrates the complexity of developing an ex vivo model and may explain why only Aquacel, amongst the five commercial wound dressings, has been previously tested ex vivo (Table S2). In those ex vivo models, Aquacel has shown varying efficacy. Notably, findings by Doherty et al. highlight that intimate contact between the dressing and the biofilm is crucial for antimicrobial activity [19]. Areas with stitching were associated with the presence of viable bacteria, as these regions created dead space, allowing bacteria within the biofilm to persist and evade the treatment. Rippon et al. observed a reduction in the number of viable *S. aureus* recovered from preformed biofilms on porcine skin treated with Aquacel, although the results showed considerable variability [23]. Similarly, Roche et al. reported that these dressings had minimal impact on biofilms in their models [20]. Their findings further suggested that the depth of bacterial penetration into the wound tissue may influence treatment effectiveness, which is consistent with our results.

This knowledge highlights the need to validate methods across dressing types due to variations in design, form, and mechanisms of action. Understanding the physicochemical and biological properties of dressings is crucial for interpreting these results. Comparing novel wound dressings with commercially available dressings offers a good approach, as they have been tested in different models and have proven clinical applications (Table S2). However, depending on the dressing's properties and mode of action, in vitro and ex vivo models may not reveal true antimicrobial efficacy, as for example Bactigras showed modest in vitro activity but was effective in vivo and in clinical trials [61] and Sorbact showed no effects in our developed assays, which contrasts with some positive clinical reports [62, 63].

These results show that the one‐model‐fits‐all approach is inadequate. Whilst the experiment offers robust initial screening, it may not detect efficacy in more complex, biorelevant models. Ex vivo models are not fully reliable, as they cannot replicate living organisms, making animal studies and clinical trials essential. However, in vitro and ex vivo models remain valuable for assessing the safety, efficacy, and enabling cost‐effective comparisons of novel wound dressings; therefore, their importance should not be underestimated. Looking ahead, our methodology has strong potential for adaptation to other novel wound dressing materials other than ES. For example, it would be possible to test stimuli‐responsive materials [64], expanding the in vitro method and ex vivo model applicability beyond conventional antimicrobial wound dressings. By integrating dynamic environmental factors, such as pH changes or enzymatic activity, future studies could leverage these models to evaluate the responsiveness of advanced wound healing materials. These conditions are particularly easy to modify in the in vitro method but are extremely relevant for mimicking real wound conditions. This perspective opens exciting opportunities for optimising wound care strategies and advancing the development of next‐generation bioactive wound dressings.

## 5 Conclusions

A rapid and economical in vitro assay and biorelevant ex vivo wound biofilm inhibition models on porcine skin were successfully developed to test the antibacterial efficacy of wound dressings and validated using different commercial antimicrobial wound dressings and an experimental ES CAM‐loaded fibre mat. Our results confirmed the importance of validating novel antimicrobial activity testing methods on a diverse range of wound dressings to understand the limitations of the assay.

Our in vitro assay offers cost‐effective screening of antimicrobial wound dressings whilst ensuring their sterility. As the testing conditions play a key role in revealing antibacterial activity in vitro, biorelevance was sought to ensure better translatability in our model. Still, the ex vivo wound biofilm model provides a more biorelevant, in vivo‐like testing system. Although biopsy wounds better mimic chronic wound conditions, even simpler skin wound biofilm models still yield reproducible antimicrobial efficacy results for wound dressings. Since in vitro and ex vivo findings may differ, both are necessary to identify inconsistencies. These complementary methods provide a comprehensive understanding of antimicrobial mechanisms, aiding clinicians in assessing wound dressings for the treatment of infections. Future studies will reveal the potential uses of our developed in vitro and ex vivo models for testing stimuli‐responsive materials, identifying strategies for mimicking real wound conditions, and enabling biorelevant testing.

## Author Contributions

**Karin Kogermann:** conceptualization. **Kaisa Põhako‐Palu, Liis Preem, Kelli Randmäe, Marta Putrinš:** formal analysis. **Karin Kogermann:** funding acquisition. **Kaisa Põhako‐Palu, Liis Preem, Kelli Randmäe:** investigation. **Kaisa Põhako‐Palu, Liis Preem, Kelli Randmäe, Marta Putrinš, Külli Kingo, Tanel Tenson, Karin Kogermann:** methodology. **Karin Kogermann:** project administration. **Külli Kongo, Tanel Tenson, Karin Kogermann:** resources. **Marta Putrinš, Külli Kingo, Tanel Tenson, Karin Kogermann:** supervision. **Kaisa Põhako‐Palu, Liis Preem, Kelli Randmäe:** visualisation. **Kaisa Põhako‐Palu:** writing – original draft. **Kaisa Põhako‐Palu, Liis Preem, Kelli Randmäe, Marta Putrinš, Külli Kingo, Tanel Tenson, Karin Kogermann:** writing – review and editing.

## Conflicts of Interest

The authors declare no conflicts of interest.

## Supporting information


**Data S1:** Supporting Information.

## Data Availability

The authors have nothing to report.

## References

[1] M. Olsson , K. Järbrink , U. Divakar , et al., “The Humanistic and Economic Burden of Chronic Wounds: A Systematic Review,” Wound Repair and Regeneration 27, no. 1 (2019): 114–125, 10.1111/wrr.12683. 30362646

[2] M. Malone , T. Bjarnsholt , A. J. McBain , et al., “The Prevalence of Biofilms in Chronic Wounds: A Systematic Review and Meta‐Analysis of Published Data,” Journal of Wound Care 26, no. 1 (2017): 20–25, 10.12968/jowc.2017.26.1.20. 28103163

[3] M. Falcone , B. De Angelis , F. Pea , et al., “Challenges in the Management of Chronic Wound Infections,” Journal of Global Antimicrobial Resistance 26 (2021): 140–147, 10.1016/j.jgar.2021.05.010. 34144200

[4] H. Q. Tran , S. M. S. Shahriar , Z. Yan , and J. Xie , “Recent Advances in Functional Wound Dressings,” Advances in Wound Care 12, no. 7 (2023): 399–427, 10.1089/wound.2022.0059. 36301918 PMC10125407

[5] A. J. Cunliffe , P. D. Askew , I. Stephan , et al., “How Do we Determine the Efficacy of an Antibacterial Surface? A Review of Standardised Antibacterial Material Testing Methods,” Antibiotics 10, no. 9 (2021): 1069, 10.3390/antibiotics10091069. 34572650 PMC8472414

[6] I. C. Thaarup and T. Bjarnsholt , “Current In Vitro Biofilm‐Infected Chronic Wound Models for Developing New Treatment Possibilities,” Advances in Wound Care 10, no. 2 (2021): 91–102, 10.1089/wound.2020.1176. 32496982

[7] E. Jordana‐Lluch , V. Garcia , A. D. H. Kingdon , et al., “A Simple Polymicrobial Biofilm Keratinocyte Colonization Model for Exploring Interactions Between Commensals, Pathogens and Antimicrobials,” Frontiers in Microbiology 11 (2020): 291, 10.3389/fmicb.2020.00291. 32161578 PMC7054238

[8] E. Lazarus , A. S. Meyer , K. Ikuma , and I. V. Rivero , “Three Dimensional Printed Biofilms: Fabrication, Design and Future Biomedical and Environmental Applications,” Microbial Biotechnology 17, no. 1 (2024): e14360, 10.1111/1751-7915.14360. 38041693 PMC10832517

[9] K. Lorenz , L. Preem , K. Sagor , M. Putrinš , T. Tenson , and K. Kogermann , “Development of In Vitro and Ex Vivo Biofilm Models for the Assessment of Antibacterial Fibrous Electrospun Wound Dressings,” Molecular Pharmaceutics 20, no. 2 (2023): 1230–1246, 10.1021/acs.molpharmaceut.2c00902. 36669095 PMC9907351

[10] H. K. N. Vyas , B. Xia , and A. Mai‐Prochnow , “Clinically Relevant In Vitro Biofilm Models: A Need to Mimic and Recapitulate the Host Environment,” Biofilms 4 (2022): 100069, 10.1016/j.bioflm.2022.100069. PMC9782257 36569981

[11] C. Wiegand , M. Abel , P. Ruth , P. Elsner , and U. C. Hipler , “In Vitro Assessment of the Antimicrobial Activity of Wound Dressings: Influence of the Test Method Selected and Impact of the pH,” Journal of Materials Science: Materials in Medicine 26, no. 1 (2015): 18, 10.1007/s10856-014-5343-9. 25578697 PMC4325976

[12] D. D. Castelo‐Branco , B. R. Amando , C. J. Ocadaque , et al., “Mini‐Review: From In Vitro to Ex Vivo Studies: An Overview of Alternative Methods for the Study of Medical Biofilms,” Biofouling 36 (2020): 1129–1148, 10.1080/08927014.2020.1859499. 33349038

[13] S. Kadam , S. Nadkarni , J. Lele , S. Sakhalkar , P. Mokashi , and K. S. Kaushik , “Bioengineered Platforms for Chronic Wound Infection Studies: How Can we Make Them More Human‐Relevant?,” Frontiers in Bioengineering and Biotechnology 7 (2019): 418, 10.3389/fbioe.2019.00418. 31921821 PMC6923179

[14] P. Chen , E. A. Sebastian , S. L. R. Karna , and K. P. Leung , “Development of a Stringent Ex Vivo‐Burned Porcine Skin Wound Model to Screen Topical Antimicrobial Agents,” Antibiotics 13, no. 12 (2024): 1159, 10.3390/antibiotics13121159. 39766550 PMC11672622

[15] M. Å. Andersson , L. B. Madsen , A. Schmidtchen , and M. Puthia , “Development of an Experimental Ex Vivo Wound Model to Evaluate Antimicrobial Efficacy of Topical Formulations,” International Journal of Molecular Sciences 22, no. 9 (2021): 5045, 10.3390/ijms22095045. 34068733 PMC8126222

[16] Q. Yang , P. L. Phillips , E. M. Sampson , et al., “Development of a Novel Ex Vivo Porcine Skin Explant Model for the Assessment of Mature Bacterial Biofilms,” Wound Repair and Regeneration 21, no. 5 (2013): 704–714, 10.1111/wrr.12074. 23927831

[17] J. h. Hwang , H. Jeong , N. Lee , et al., “Ex Vivo Live Full‐Thickness Porcine Skin Model as a Versatile In Vitro Testing Method for Skin Barrier Research,” International Journal of Molecular Sciences 22, no. 2 (2021): 657, 10.3390/ijms22020657. 33440780 PMC7827261

[18] G. E. Flaten , Z. Palac , A. Engesland , J. Filipović‐Grčić , Ž. Vanić , and N. Škalko‐Basnet , “In Vitro Skin Models as a Tool in Optimization of Drug Formulation,” European Journal of Pharmaceutical Sciences 75 (2015): 10–24, 10.1016/j.ejps.2015.02.018. 25746955

[19] C. Doherty , C. V. Byrne , S. Baqader , C. El‐Chami , A. J. McBain , and H. A. Thomason , “Anti‐Biofilm Effects and Healing Promotion by Silver Oxynitrate‐Based Dressings,” Scientific Reports 13, no. 1 (2023): 2014, 10.1038/s41598-022-26856-x. 36737464 PMC9898495

[20] E. D. Roche , E. J. Woodmansey , Q. Yang , D. J. Gibson , H. Zhang , and G. S. Schultz , “Cadexomer Iodine Effectively Reduces Bacterial Biofilm in Porcine Wounds Ex Vivo and In Vivo,” International Wound Journal 16, no. 3 (2019): 674–683, 10.1111/iwj.13080. 30868761 PMC6850490

[21] V. De Maesschalck , D. Gutiérrez , J. Paeshuyse , Y. Briers , G. Vande Velde , and R. Lavigne , “A Bioluminescence‐Based Ex Vivo Burn Wound Model for Real‐Time Assessment of Novel Phage‐Inspired Enzybiotics,” Pharmaceutics 14, no. 12 (2022): 2553, 10.3390/pharmaceutics14122553. 36559047 PMC9781546

[22] G. M. D. M. Guedes , R. M. Pinheiro , A. S. Freitas , et al., “Ex Vivo Wound Model on Porcine Skin for the Evaluation of the Antibiofilm Activity of Polyhexamethylene Biguanide and Ciprofloxacin,” Letters in Applied Microbiology 76, no. 3 (2023): ovad031, 10.1093/lambio/ovad031. 36841231

[23] M. G. Rippon , A. A. Rogers , L. Sellars , K. M. Styles , and S. Westgate , “Effectiveness of a Non‐Medicated Wound Dressing on Attached and Biofilm Encased Bacteria: Laboratory and Clinical Evidence,” Journal of Wound Care 27, no. 3 (2018): 146–155, 10.12968/jowc.2018.27.3.146. 29509112

[24] M. Portugal‐Cohen , D. Cohen , R. Kohen , and M. Oron , “Exploitation of Alternative Skin Models From Academia to Industry: Proposed Functional Categories to Answer Needs and Regulation Demands,” Frontiers in Physiology 14 (2023): 1215266, 10.3389/fphys.2023.1215266. 37334052 PMC10272927

[25] T. Bjarnsholt , M. Whiteley , K. P. Rumbaugh , P. S. Stewart , P. Ø. Jensen , and N. Frimodt‐Møller , “The Importance of Understanding the Infectious Microenvironment,” Lancet Infectious Diseases 22, no. 3 (2022): e88–e92, 10.1016/S1473-3099(21)00122-5. 34506737 PMC9190128

[26] S. Ud‐Din and A. Bayat , “Non‐Animal Models of Wound Healing in Cutaneous Repair: In Silico, In Vitro, Ex Vivo, and In Vivo Models of Wounds and Scars in Human Skin,” Wound Repair and Regeneration 25, no. 2 (2017): 164–176, 10.1111/wrr.12513. 28120405

[27] E. Pinho , L. Magalhães , M. Henriques , and R. Oliveira , “Antimicrobial Activity Assessment of Textiles: Standard Methods Comparison,” Annales de Microbiologie 61, no. 3 (2011): 493–498, 10.1007/s13213-010-0163-8.

[28] L. Cleaver and J. A. Garnett , “How to Study Biofilms: Technological Advancements in Clinical Biofilm Research,” Frontiers in Cellular and Infection Microbiology 13 (2023): 1335389, 10.3389/fcimb.2023.1335389. 38156318 PMC10753778

[29] A. Gefen , P. Alves , D. Beeckman , et al., “How Should Clinical Wound Care and Management Translate to Effective Engineering Standard Testing Requirements From Foam Dressings? Mapping the Existing Gaps and Needs,” Advances in Wound Care 13, no. 1 (2024): 34–52, 10.1089/wound.2021.0173. 35216532 PMC10654650

[30] B. Cullen and A. Gefen , “The Biological and Physiological Impact of the Performance of Wound Dressings,” International Wound Journal 20, no. 4 (2023): 1292–1303, 10.1111/iwj.13960. 36110054 PMC10031231

[31] L. Preem , M. Mahmoudzadeh , M. Putrinš , et al., “Interactions Between Chloramphenicol, Carrier Polymers, and Bacteria–Implications for Designing Electrospun Drug Delivery Systems Countering Wound Infection,” Molecular Pharmaceutics 14, no. 12 (2017): 4417–4430, 10.1021/acs.molpharmaceut.7b00524. 29099601

[32] L. Preem , F. Bock , M. Hinnu , et al., “Monitoring of Antimicrobial Drug Chloramphenicol Release From Electrospun Nano‐ and Microfiber Mats Using UV Imaging and Bacterial Bioreporters,” Pharmaceutics 11, no. 9 (2019): 487, 10.3390/pharmaceutics11090487. 31546922 PMC6781501

[33] D. Parsons , K. Meredith , V. J. Rowlands , D. Short , D. G. Metcalf , and P. G. Bowler , “Enhanced Performance and Mode of Action of a Novel Antibiofilm Hydrofiber Wound Dressing,” BioMed Research International 2016 (2016): 1–14, 10.1155/2016/7616471. PMC5136405 27990437

[34] “AQUACEL AG+ Extra Dressings,” ConvaTec, accessed February 9, 2025, https://www.convatec.com/en‐sg/products/advanced‐wound‐care/wound‐type/pc‐wound‐diabetic‐foot‐ulcers/21603750‐5757‐4a85‐a075‐465347dbfccb/.

[35] K. Ziegler , R. Görl , J. Effing , et al., “Reduced Cellular Toxicity of a New Silver‐Containing Antimicrobial Dressing and Clinical Performance in Non‐Healing Wounds,” Skin Pharmacology and Physiology 19, no. 3 (2006): 140–146, 10.1159/000092594. 16612141

[36] Hartmann Group , “Atrauman Ag,” accessed February 9, 2025, https://www.hartmann.info/en‐au/products/wound‐management/contact‐layers/impregnated‐contact‐layers/atrauman®‐ag.

[37] J. K. Andrews , I. A. Buchan , and M. Horlington , “An Experimental Evaluation of a Chlorhexidine Medicated Tulle Gras Dressing,” Journal of Hospital Infection 3, no. 2 (1982): 149–157, 10.1016/0195-6701(82)90007-X. 6181132

[38] “Bactigras PPL,” accessed February 9, 2025, https://www.smith‐nephew.com/en/health‐care‐professionals/products/advanced‐wound‐management/bactigras‐ppl.

[39] J. Husmark , B. Morgner , Y. B. Susilo , and C. Wiegand , “Antimicrobial Effects of Bacterial Binding to a Dialkylcarbamoyl Chloride‐Coated Wound Dressing: An In Vitro Study,” Journal of Wound Care 31, no. 7 (2022): 560–570, 10.12968/jowc.2022.31.7.560. 35797260

[40] “Sorbact Compress—Sorbact for Healthcare Professionals,” accessed February 9, 2025, https://sorbact.com/product/sorbact‐compress/.

[41] S. Fumarola , M. Butcher , P. Cooper , et al., “A Clinical Audit of Suprasorb X + PHMB,” Wounds UK 6, no. 3 (2010): 78–87.

[42] “L&R Global: Suprasorb X + PHMB,” accessed February 9, 2025, https://www.lohmann‐rauscher.com/en/products/wound‐care/modern‐wound‐care/suprasorb‐x‐phmb/.

[43] K. Põhako‐Palu , K. Lorenz , K. Randmäe , et al., “In Vitro Experimental Conditions and Tools Can Influence the Safety and Biocompatibility Results of Antimicrobial Electrospun Biomaterials for Wound Healing,” PLoS One 19, no. 7 (2024): e0305137, 10.1371/journal.pone.0305137. 38950036 PMC11216574

[44] Š. Zupančič , L. Preem , J. Kristl , et al., “Impact of PCL Nanofiber Mat Structural Properties on Hydrophilic Drug Release and Antibacterial Activity on Periodontal Pathogens,” European Journal of Pharmaceutical Sciences 122 (2018): 347–358, 10.1016/j.ejps.2018.07.024. 30017845

[45] Y. Yang , Y. Du , J. Zhang , H. Zhang , and B. Guo , “Structural and Functional Design of Electrospun Nanofibers for Hemostasis and Wound Healing,” Advanced Fiber Materials 4, no. 5 (2022): 1027–1057, 10.1007/s42765-022-00178-z.

[46] X. Z. Li , D. M. Livermore , and H. Nikaido , “Role of Efflux Pump(s) in Intrinsic Resistance of *Pseudomonas aeruginosa* : Resistance to Tetracycline, Chloramphenicol, and Norfloxacin,” Antimicrobial Agents and Chemotherapy 38, no. 8 (1994): 1732–1741, 10.1128/AAC.38.8.1732. 7986003 PMC284630

[47] V. Puca , R. Z. Marulli , R. Grande , et al., “Microbial Species Isolated From Infected Wounds and Antimicrobial Resistance Analysis: Data Emerging From a Three‐Years Retrospective Study,” Antibiotics 10, no. 10 (2021): 1162, 10.3390/antibiotics10101162. 34680743 PMC8532735

[48] H. C. Ring , P. Theut Riis , L. Bay , K. Kallenbach , T. Bjarnsholt , and G. B. E. Jemec , “Haematoxylin and Eosin Staining Identifies Medium to Large Bacterial Aggregates With a Reliable Specificity: A Comparative Analysis of Follicular Bacterial Aggregates in Axillary Biopsies Using Peptide Nucleic Acid‐Fluorescence in Situ Hybridization and Haematoxylin and Eosin Staining,” Experimental Dermatology 26, no. 10 (2017): 943–945, 10.1111/exd.13338. 28266778

[49] S. D. Hong , H. J. Dhong , S. K. Chung , H. Y. Kim , J. Park , and S. Y. Ha , “Hematoxylin and Eosin Staining for Detecting Biofilms: Practical and Cost‐Effective Methods for Predicting Worse Outcomes After Endoscopic Sinus Surgery,” Clinical and Experimental Otorhinolaryngology 7, no. 3 (2014): 193, 10.3342/ceo.2014.7.3.193. 25177435 PMC4135155

[50] P. Chadwick and K. Ousey , “Bacterial‐Binding Dressings in the Management of Wound Healing and Infection Prevention: A Narrative Review,” Journal of Wound Care 28, no. 6 (2019): 370–382, 10.12968/jowc.2019.28.6.370. 31166862

[51] Q. Song , S. Y. Chan , Z. Xiao , et al., “Contact‐Killing Antibacterial Mechanisms of Polycationic Coatings: A Review,” Progress in Organic Coating 188 (2024): 108214, 10.1016/j.porgcoat.2024.108214.

[52] Y. Ilg and J. Kreyenschmidt , “Effects of Food Components on the Antimicrobial Activity of Polypropylene Surfaces Containing Silver Ions (Ag^+^),” International Journal of Food Science and Technology 46, no. 7 (2011): 1469–1476, 10.1111/j.1365-2621.2011.02641.x.

[53] G. Akrong , A. Chauzy , V. Aranzana‐Climent , et al., “A New Pharmacokinetic‐Pharmacodynamic Model to Characterize the Inoculum Effect of *Acinetobacter baumannii* on Polymyxin B in Vitro,” Antimicrobial Agents and Chemotherapy 66, no. 1 (2022): e01789‐21, 10.1128/AAC.01789-21. 34780268 PMC8765416

[54] L. Rio‐Marques , A. Hartke , and A. Bizzini , “The Effect of Inoculum Size on Selection of in Vitro Resistance to Vancomycin, Daptomycin, and Linezolid in Methicillin‐Resistant *Staphylococcus aureus* ,” Microbial Drug Resistance 20, no. 6 (2014): 539–543, 10.1089/mdr.2014.0059. 25010140

[55] M. R. Loffredo , F. Savini , S. Bobone , et al., “Inoculum Effect of Antimicrobial Peptides,” Proceedings of the National Academy of Sciences 118, no. 21 (2021): e2014364118, 10.1073/pnas.2014364118. PMC8166072 34021080

[56] S. M. Wirth , A. J. Bertuccio , F. Cao , G. V. Lowry , and R. D. Tilton , “Inhibition of Bacterial Surface Colonization by Immobilized Silver Nanoparticles Depends Critically on the Planktonic Bacterial Concentration,” Journal of Colloid and Interface Science 467 (2016): 17–27, 10.1016/j.jcis.2015.12.049. 26771749

[57] P. Aramwit , P. Muangman , N. Namviriyachote , and T. Srichana , “In Vitro Evaluation of the Antimicrobial Effectiveness and Moisture Binding Properties of Wound Dressings,” International Journal of Molecular Sciences 11, no. 8 (2010): 2864–2874, 10.3390/ijms11082864. 21152279 PMC2996738

[58] F. Song , H. Koo , and D. Ren , “Effects of Material Properties on Bacterial Adhesion and Biofilm Formation,” Journal of Dental Research 94, no. 8 (2015): 1027–1034, 10.1177/0022034515587690. 26001706

[59] R. A. G. Da Silva , I. Afonina , and K. A. Kline , “Eradicating Biofilm Infections: An Update on Current and Prospective Approaches,” Current Opinion in Microbiology 63 (2021): 117–125, 10.1016/j.mib.2021.07.001. 34333239

[60] M. E. Levison and J. H. Levison , “Pharmacokinetics and Pharmacodynamics of Antibacterial Agents,” Infectious Disease Clinics of North America 23, no. 4 (2009): 791–815, 10.1016/j.idc.2009.06.008. 19909885 PMC3675903

[61] J. C. Lawrence , “The Treatment of Small Burns With a Chlorhexidine‐Medicated Tulle Gras,” Burns 3, no. 4 (1977): 239–244, 10.1016/0305-4179(77)90049-3.

[62] S. Haycocks and P. Chadwick , “Use of DACC‐ Coated Dressings in Diabetic Foot Ulcers: A Case Series,” Diabetic Foot Journal 14, no. 3 (2011): 133–137.

[63] A. M. Nielsen and A. Andriessen , “Prospective Cohort Study on Surgical Wounds Comparing a Polyhexanide‐Containing Biocellulose Dressing With a Dialkyl‐Carbamoyl‐Chloride–Containing Hydrophobic Dressing,” Advances in Skin & Wound Care 25, no. 9 (2012): 409–413, 10.1097/01.ASW.0000419406.29700.ef. 22914037

[64] D. Rybak , J. Du , P. Nakielski , et al., “NIR‐Light Activable 3D Printed Platform Nanoarchitectured With Electrospun Plasmonic Filaments for on Demand Treatment of Infected Wounds,” Advanced Healthcare Materials 14 (2024): 1–17, 10.1002/adhm.202404274. PMC11874648 39722151

