# Supplementary material for: Development of Rapid and Economic In Vitro Assay and Biorelevant Ex Vivo Biofilm Inhibition Wound Model to Test the Antibacterial Efficacy of Wound Dressings
Source: Wound Repair Regen. 2025 Aug 18;33(4):e70080. doi: 10.1111/wrr.70080 (PMC12361873; doi:10.1111/wrr.70080)
Supplement: Supplementary file 1 — Data S1: Supporting Information. [file WRR-33-0-s001.docx]

Supplementary material

**Development of rapid and economic *in vitro* assay and biorelevant *ex vivo* biofilm inhibition wound model to test the antibacterial efficacy of wound dressings**

Kaisa Põhako-Palu^1^, Liis Preem^1^, Kelli Randmäe^1^, Marta Putrinš^1^, Külli Kingo^2^, Tanel Tenson^3^, Karin Kogermann^1*^

^1^ Institute of Pharmacy, University of Tartu, Estonia

^2^ Dermatology Clinic, Tartu University Hospital, Estonia

^3^ Institute of Technology, University of Tartu, Estonia

**Corresponding Author and requests for reprints**

Karin Kogermann; Mailing address: Institute of Pharmacy, University of Tartu, Nooruse 1, 50411 Tartu, Estonia; Tel.: +372 737 5281; Fax: +372 737 5289; Mobile phone: +372 56 509 455; E-mail address: [karin.kogermann@ut.ee](mailto:karin.kogermann@ut.ee); ORCID: 0000-0002-6813-4828

**Keywords:** wound models, biofilm, chronic wounds, electrospun materials

ASTM E2180-18 *in vitro* assay

The ASTM E2180-18 assay, a standard method for determining the activity of incorporated antimicrobial agent(s) in polymeric or hydrophobic materials, was used to test the Atrauman and Sorbact dressings. As we saw in our developed *in vitro* assay, inoculum size is an important factor in determining the efficacy of a dressing against bacteria. Hence, we wanted to determine how these dressings behave in this assay when challenged with different inoculum densities. For Sorbact, the inoculum levels of *E. coli* and *S. aureus* were reduced below 100 CFU/cm^2^, but the dressing did not show any antimicrobial activity under these conditions. By comparison, Atrauman was active even when the inocula of all tested bacteria were increased (Figure S1).


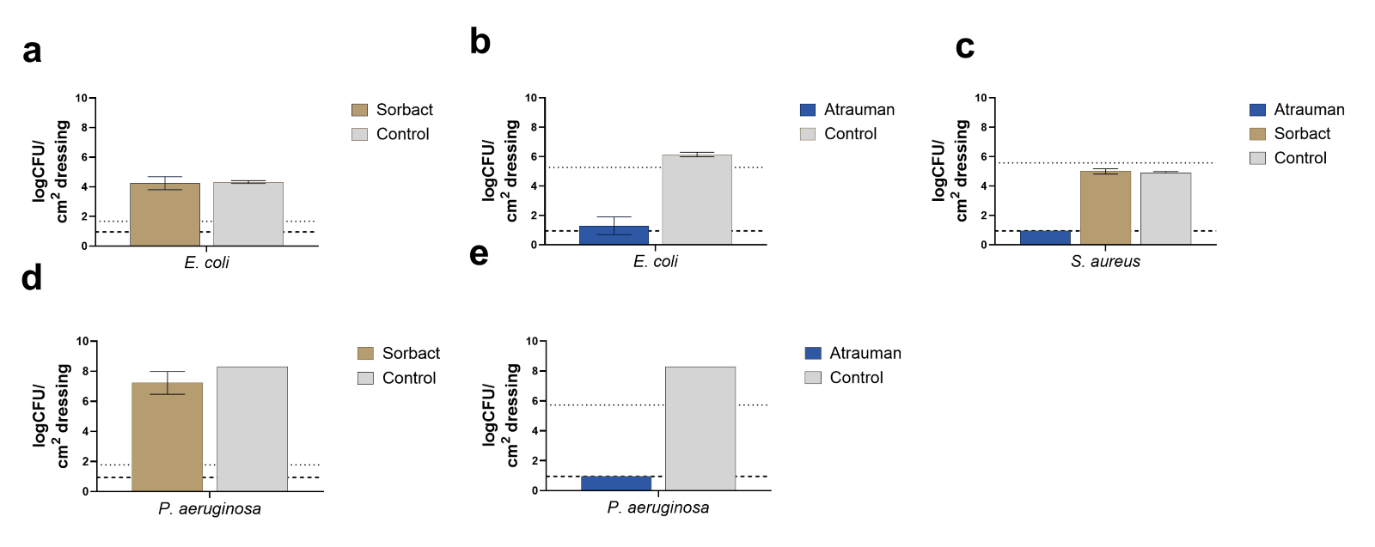
**Figure S1.** Mean and standard deviation (N=3) of the log-transformed number of *E. coli* DSM1103 (a and b), *S. aureus* DSM2569 (c) or *P. aeruginosa* DSM1117 (d and e) colony-forming units (CFUs) detected on Atrauman and/or Sorbact dressings, and in a control in ASTM E218-18 assay with different initial inoculum sizes. The dotted line represents the number of bacteria in the initial inoculum (logCFU/cm^2^ of dressing) and the dashed line represents the detection limit.

Detection of planktonic bacteria from *ex vivo* wound biofilm inhibition models

The detection of planktonic bacteria from *ex vivo* wound biofilm inhibition models (BIM-T, WBIM-T, WBIM-I) was performed as described in the Method section (*Ex vivo* wound biofilm inhibition models on porcine skin). The levels of planktonic bacteria were detected separately from the dressings and from the porcine skin. For the analysis, the CFUs were summarised. Summarized detected CFU levels of planktonic bacteria by model types are shown in Figure S2.


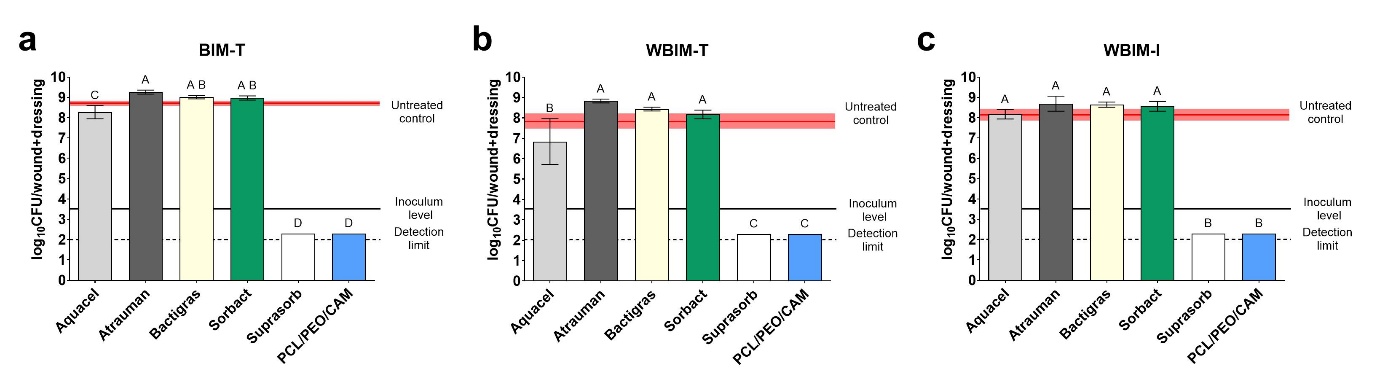
**Figure S2.** Mean of the log-transformed total *E. coli* planktonic bacterial growth on both the skin and the dressing in BIM (a), WBIM-T (b) and WBIM-I (c). The red line marks the mean untreated control bacterial levels with SD (pink area), solid black line inoculum level 5x10^3^ and dotted line the detection limit of 200 CFU. The data for commercial wound dressings are represented as the mean of three biological replicates (N=3), and the data for ES materials are represented as the mean of nine biological replicates (N=9). Key: BIM – biofilm inhibition model; CAM – chloramphenicol; CFU – colony forming unit; PCL – polycaprolactone; PEO – polyethylene oxide; WBIM – wound biofilm inhibition model; T - on top; I - inside. The GraphPad compact letter display feature was used to indicate statistically significant differences between the dressings in pairwise comparisons. In each model, no statistically significant differences were observed between the two dressings sharing a letter.

Detection of bacteria on wound dressings

The number of bacteria growing on the wound dressing detected from *ex vivo* (wound) biofilm inhibition models was performed as described in the Methods section (*Ex vivo* wound biofilm inhibition models on porcine skin). The results are shown in Figure S3. In each model, bacterial growth on skin treated with Aquacel, Atrauman, Bactigras and Sorbact was detected at the level of 10^8^ CFU. Among the commercial dressings, the least number of bacteria on the skin was detected when treated with Suprasorb. In all models, using PCL/PEO/CAM for treatment, the detected bacterial growth was near the detection limit of 200 CFU.


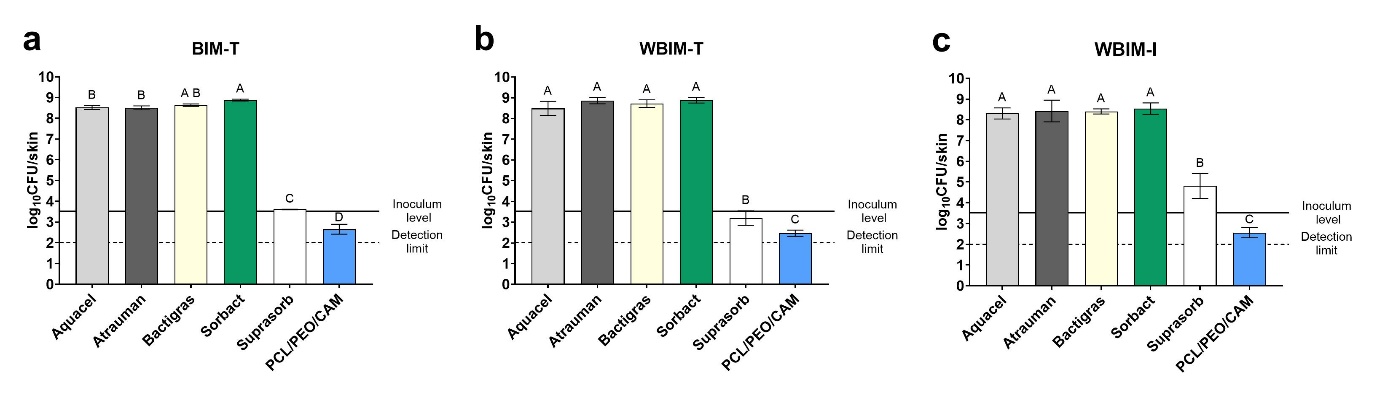
**Figure S3**. Mean of log-transformed *E. coli* bacterial growth on skin treated with wound dressings in BIM (a), WBIM-T (b) and WBIM-I (c). The solid black line represents the inoculum level of 5x10^3^ and dotted line represents the detection limit of 200 CFU. The data for commercial wound dressings are represented as the average of three biological replicates (N=3), and the data for ES materials are represented as the average of nine biological replicates (N=9). Key: BIM – biofilm inhibition model; CAM – chloramphenicol; CFU – colony forming unit; PCL – polycaprolactone; PEO – polyethylene oxide; WBIM – wound biofilm inhibition model; T - on top; I - inside. The GraphPad compact letter display feature is used to indicate statistically significant differences between dressings in pairwise comparisons. In each model, no statistically significant differences were observed between the two dressings sharing a letter.

CFU levels of modified versions of *ex vivo* wound biofilm inhibition models treated with Aquacel and Bactigras dressings

The detection of the CFU level of the dressings and skin in *ex vivo* wound biofilm inhibition models was performed as described in the Methods section (*Ex vivo* wound biofilm inhibition models on porcine skin). Additionally, 100, 200, and 300 µL of DMEM/Ham-12 medium were added to the Aquacel-treated models and 25, 50, and 75 µL to the Bactigras-treated models to enhance the antibacterial activity of antibacterial agents in the dressings. The results are shown in Figure S4. In the case of Aquacel, no change was observed in BIM-T (Figure S4a), but some slight changes were observed in WBIM-T and WBIM-I (Figures S4b and S4e). Changes in Bactigras antibacterial activity were not observed in any models (Figures S4d, S4e and S4f). Table S1 shows separately the CFU levels detected on both the Aquacel dressing and the skin for each model and its modification. In some cases, a very large variability was observed between the three parallels (for example, the WBIM-I dressing detected CFUs).


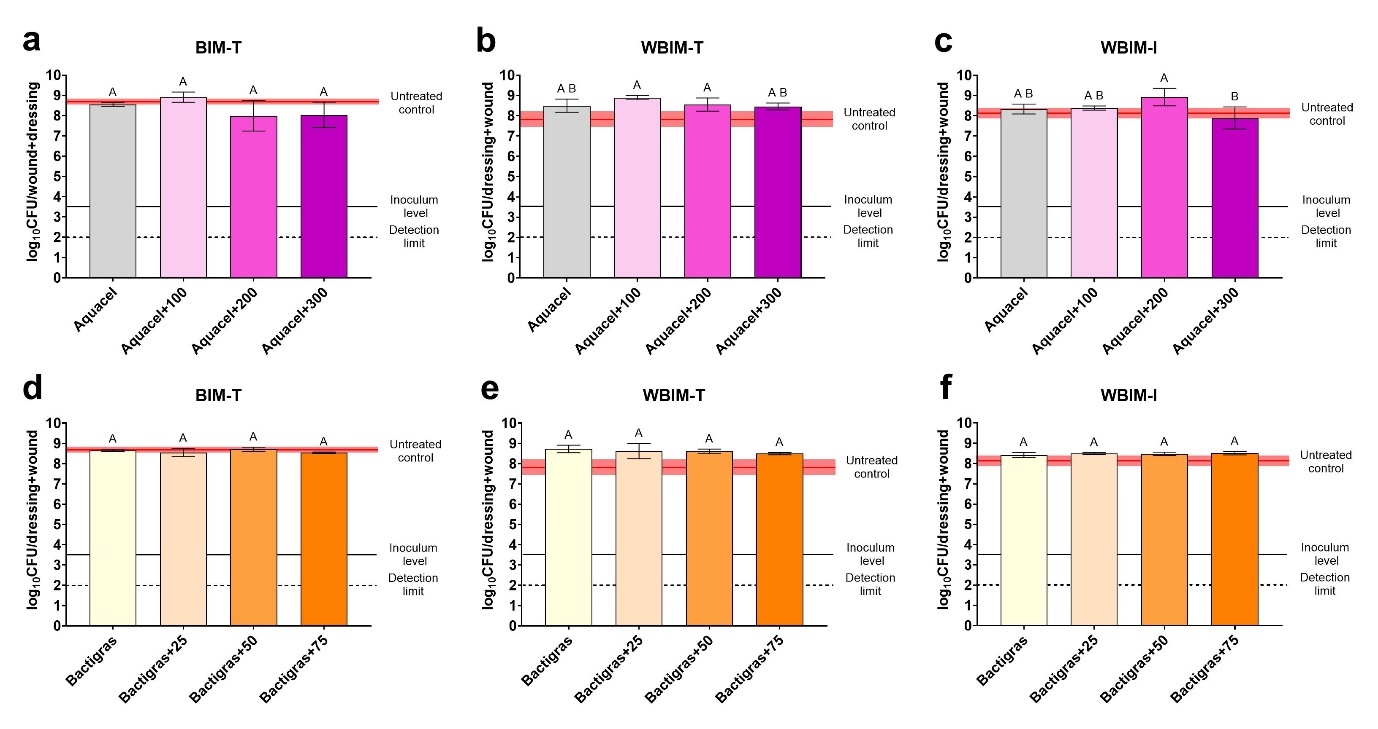


**Figure S4.** Mean of the log-transformed total *E. coli* bacterial growth on both the skin and the Aquacel and Bactigras dressings in BIM (a), WBIM-T (b) and WBIM-I (c). The red line marks the mean untreated control bacterial levels with SD (pink area), solid black line inoculum level of 5x10^3^ and dotted line indicates the detection limit of 100 CFU. The data are represented as the average of three biological replicates (N=3). Key: BIM – biofilm inhibition model; CFU – colony forming unit, WBIM – wound biofilm inhibition model; T - on top; I - inside. The GraphPad compact letter display feature is used to indicate statistically significant differences between dressings in pairwise comparisons. In each model, no statistically significant differences were observed between the two dressings sharing a letter.

**Table S1.** Detected CFU levels in different *ex vivo* models treated with Aquacel dressing and the amount of medium used for irrigation.

| **Model** | **Amount of medium(µL)** | **Dressing CFU** | | | **Skin CFU** | | |
| --- | --- | --- | --- | --- | --- | --- | --- |
| BIM-T | - | 3.47E+07 | 3.00E+07 | 1.60E+07 | 2.87E+08 | 4.27E+08 | 3.00E+08 |
|  | 100 | 4.80E+07 | 2.07E+05 | 4.27E+06 | 5.20E+08 | 1.60E+09 | 6.00E+08 |
|  | 200 | 4.00E+07 | 2.47E+07 | 1.53E+06 | 5.93E+07 | 5.33E+08 | 1.60E+07 |
|  | 300 | 3.13E+06 | 4.07E+06 | 3.80E+06 | 1.73E+07 | 2.47E+08 | 2.47E+08 |
| WBIM-T | - | 2.00E+06 | 3.53E+06 | 1.13E+07 | 2.33E+08 | 7.33E+08 | 1.67E+08 |
|  | 100 | 6.67E+07 | 3.47E+06 | 1.13E+06 | 7.33E+08 | 1.00E+09 | 6.00E+08 |
|  | 200 | 1.67E+08 | 1.73E+06 | 3.80E+06 | 6.67E+08 | 2.80E+08 | 1.93E+08 |
|  | 300 | 3.47E+07 | 9.33E+05 | 1.00E+07 | 3.20E+08 | 3.67E+08 | 1.73E+08 |
| WBIM-I | - | 1.67E+07 | 1.40E+07 | 4.67E+06 | 2.80E+08 | 1.00E+08 | 3.00E+08 |
|  | 100 | 8.67E+06 | 7.33E+03 | 9.33E+03 | 2.93E+08 | 2.47E+08 | 1.87E+08 |
|  | 200 | 3.33E+07 | 6.67E+01 | 1.80E+06 | 2.00E+09 | 2.87E+08 | 1.00E+09 |
|  | 300 | 3.67E+06 | 1.07E+06 | 1.40E+04 | 1.67E+08 | 1.73E+07 | 1.53E+08 |

Key: BIM – biofilm inhibition model; CFU – colony forming unit, WBIM – wound biofilm inhibition model; T - on top; I - inside.

Literature overview of models and clinical trials to test antibacterial efficacy of commercially available wound dressings used in this study

A description of the models and clinical trials found in the literature to test the efficacy of commercial wound dressings used to validate the models in this study are presented in Table S2.

**Table S2**. A literature overview of the methods and models previously used to test the commercially available antimicrobial wound dressings tested in this study.

| **Dressing** | ***In vitro* methods** | ***Ex vivo* methods** | ***In vivo* methods** | **Clinical trials** |
| --- | --- | --- | --- | --- |
| Atrauman | Silicone disc based *in vitro* *S. aureus* biofilm model^1^. *In vitro* standard ASTM 2180 method^2^. | No data found | Used as a control in burn wound experiments on rats^3^. | Clinical trial on patients with  non-healing wounds^2^ and different ulcers^4–6^. Has been used in clinical trials in combination with NWSP^7,8^. |
| Aquacel | *P. aeruginosa* and *S. aureus* *in vitro* biofilm assay on gyclopore membranes^9^, multi-well plate method and CDC reactor model^10^. Calorimetric *in vitro* assay testing efficacy against *S. aureus*^11^. Shake flask method testing efficacy against *S. aureus*^12^. Simulated skin with TSA agar contact plate for testing efficacy against *Klebsiella pneumoniae* and MRSA co-culture^13^ or MRSA alone^14^. Filter disk-based assay for testing efficacy against antibiotic resistant *P. aeruginosa*, *Candida albicans* and MRSA, *Klebsiella pneumoniae* and *S. aureus*^15^. | *Ex vivo* model on porcine skin with *P. aeruginosa* and/or *S. aureus* biofilm^9,10,16^. | Porcine model without infection testing interference of the additional anti-biofilm excipients on normal wounds^17^. Porcine model with mixed infection containing *S. aureus* and *P. aeruginosa* for testing antimicrobial efficacy^16^. Infected wound model on rabbits and on biopsy-wounded mice testing the efficacy against *S. aureus* and *P. aeruginosa*^9,18^. | Clinical study on patients with hard to heal wounds that have previously treated with PHMB^19^. Clinical trial on UK and Ireland patients with different challenging wounds for safety and efficacy evaluation^20,21^. Clinical trial on patients with chronic leg ulcerations^22^. |
| Bactigras | *S. aureus, E. coli* and *P. aeruginosa* bacterial suspension based assay^23^.  Silicone disc based *in vitro* *S. aureus* biofilm model^1^. | No data found | Animal study on guinea pigs with burn wounds infected with *S. aureus*^24^ and *E. coli*^25^. Study on rats with biopsy wounds infected with MRSA^26^. | Clinical trials on burn wound patients have shown effectiveness against *S. aureus*^24^. Bactigras dressing has been used as a control in studies investigating treatment of split-skin graft sites of burn wound patients^27,28^ and patients with ulcers^5^. |
| Sorbact | Agar diffusion test against *S. aureus* and *P. aeruginosa*, antimicrobial activity has been tested as described in the JIS L1902 standard and investigation of the sustained antimicrobial effect using continuous addition of bacterial culture onto piece of wound dressing^29^ | No data found | Animal study on pigs to test pressure transduction, wound edge contraction, blood flow and retention in negative pressure treatment^30^. | Clinical study on patients with diabetic foot ulceration^31^.  Cohort study on patients with surgical wounds^32^. Used in trial in combination with NPWT to treat heavily exudating infected wounds^33^. Randomized controlled trial to evaluate the prevention of surgical site infection in Cesarean section wounds^34^. |
| Suprasorb | Agar/gelatine 3D multispecies (*S. aureus*, *E. coli*, and *A. baumannii*) biofilm model^35^.  Biopsy-wounded artificial 3D skin model infected with *S. aureus* or *P. aeruginosa*^36^. Co-culture of HaCaT keratinocytes and *S. aureus*^37^. | No data found | No data found | Clinical trial on patients with critically colonized wounds^38^. Cohort study on patients with non-healing infected wounds^39^ and on surgical wounds^32^. Randomized controlled study on burn wound patients^40^. |

Key: 3D – three dimensional; CDC - Center for Disease Control; MRSA – Methicillin-resistant *Staphylococcus aureus*; NWSP - negative pressure wound therapy; PHMB - polyhexamethylene biguanide; TSA – tryptic soy agar.

**References**

1. Brackman, G., De Meyer, L., Nelis, H. J. & Coenye, T. Biofilm inhibitory and eradicating activity of wound care products against *Staphylococcus aureus* and *Staphylococcus epidermidis* biofilms in an *in vitro* chronic wound model. *J Appl Microbiol* **114**, 1833–1842 (2013).

2. Ziegler, K. *et al.* Reduced Cellular Toxicity of a New Silver-Containing Antimicrobial Dressing and Clinical Performance in Non-Healing Wounds. *Skin Pharmacol Physiol* **19**, 140–146 (2006).

3. Coman, C.-G. *et al.* Chitosan-Electrospun Fibers Encapsulating Norfloxacin: The Impact on the Biochemical, Oxidative and Immunological Profile in a Rats Burn Model. *IJMS* **25**, 12709 (2024).

4. Barrett, S., Rippon, M. G. & Rogers, A. A. Effectiveness of wound contact layers in enabling undisturbed wound management: a case series. *J Wound Care* **32**, 134–144 (2023).

5. Senejko, M., Pasek, J., Szajkowski, S., Cieślar, G. & Sieroń, A. Evaluation of the therapeutic efficacy of activespecialistic medical dressings in the treatment ofdecubitus. *pdia* **38**, 75–79 (2021).

6. Pasek, J., Szajkowski, S. & Cieślar, G. Application of Topical Hyperbaric Oxygen Therapy and Medical Active Dressings in the Treatment of Arterial Leg Ulcers—A Pilot Study. *Sensors* **23**, 5582 (2023).

7. Bukovcan, P., Koller, J., Hajská, M. & Záhorec, P. Clinical Experience With the Use of Negative Pressure Wound Therapy Combined With a Silver-impregnated Dressing in Mixed Wounds: A Retrospective Study of 50 Cases. *Wounds* **28**, 255–263 (2016).

8. Walczak, D. A. *et al.* Management of large chronic venous leg ulcers with negative pressure wound therapy. *NPWTJ* **4**, 17 (2017).

9. Doherty, C. *et al.* Anti-biofilm effects and healing promotion by silver oxynitrate-based dressings. *Sci Rep* **13**, 2014 (2023).

10. Rippon, M. G., Rogers, A. A., Sellars, L., Styles, K. M. & Westgate, S. Effectiveness of a non-medicated wound dressing on attached and biofilm encased bacteria: laboratory and clinical evidence. *J Wound Care* **27**, 146–155 (2018).

11. Said, J. *et al.* An in vitro test of the efficacy of an anti-biofilm wound dressing. *International Journal of Pharmaceutics* **474**, 177–181 (2014).

12. Bourdillon, K. A., Delury, C. P. & Cullen, B. M. Biofilms and delayed healing – an in vitro evaluation of silver‐ and iodine‐containing dressings and their effect on bacterial and human cells. *International Wound Journal* **14**, 1066–1075 (2017).

13. Meredith, K., Coleborn, M. M., Forbes, L. E. & Metcalf, D. G. Assessment of the Antibiofilm Performance of Silver-Containing Wound Dressings: A Dual-Species Biofilm Model. *Cureus* (2024) doi:10.7759/cureus.70086.

14. Bowler, P. G. & Parsons, D. Combatting wound biofilm and recalcitrance with a novel anti-biofilm Hydrofiber® wound dressing. *Wound Medicine* **14**, 6–11 (2016).

15. Parsons, D. *et al.* Enhanced Performance and Mode of Action of a Novel Antibiofilm Hydrofiber® Wound Dressing. *BioMed Research International* **2016**, 1–14 (2016).

16. Roche, E. D. *et al.* Cadexomer iodine effectively reduces bacterial biofilm in porcine wounds ex vivo and in vivo. *International Wound Journal* **16**, 674–683 (2019).

17. Davis, S. C. *et al.* The wound‐healing effects of a next‐generation anti‐biofilm silver Hydrofiber wound dressing on deep partial‐thickness wounds using a porcine model. *International Wound Journal* **15**, 834–839 (2018).

18. Seth, A. K. *et al.* Impact of a novel, antimicrobial dressing on in vivo, *P* *seudomonas aeruginosa* wound biofilm: Quantitative comparative analysis using a rabbit ear model. *Wound Repair Regeneration* **22**, 712–719 (2014).

19. Metcalf, D. G. & Bowler, P. G. Clinical impact of an anti-biofilm Hydrofiber dressing in hard-to-heal wounds previously managed with traditional antimicrobial products and systemic antibiotics. *Burns & Trauma* **8**, tkaa004 (2020).

20. Metcalf, D., Parsons, D. & Bowler, P. A next-generation antimicrobial wound dressing: a real-life clinical evaluation in the UK and Ireland. *J Wound Care* **25**, 132–138 (2016).

21. Metcalf, D. G., Parsons, D. & Bowler, P. G. Clinical safety and effectiveness evaluation of a new antimicrobial wound dressing designed to manage exudate, infection and biofilm. *International Wound Journal* **14**, 203–213 (2017).

22. Wilson, P., Gillen, C. & Hughes, M. A clinical case series on the effectiveness of an enhanced ionic silver hydrofiber dressing in the management of diabetic foot ulceration. **21**, (2018).

23. Holland, K. T. & Davis, W. A note on an *in vitro* test system to compare the bactericidal properties of wound dressings. *Journal of Applied Bacteriology* **59**, 61–63 (1985).

24. Lawrence, J. C. The treatment of small burns with a chlorhexidine-medicated tulle gras. *Burns* **3**, 239–244 (1977).

25. Andrews, J. K., Buchan, I. A. & Horlington, M. An experimental evaluation of a chlorhexidine medicated tulle gras dressing. *Journal of Hospital Infection* **3**, 149–157 (1982).

26. Ülkür, E., Oncul, O., Karagoz, H., Yeniz, E. & Çeliköz, B. Comparison of silver-coated dressing (Acticoat^TM^), chlorhexidine acetate 0.5% (Bactigrass®), and fusidic acid 2% (Fucidin®) for topical antibacterial effect in methicillin-resistant Staphylococci-contaminated, full-skin thickness rat burn wounds. *Burns* **31**, 874–877 (2005).

27. Muangman, P., Nitimonton, S. & Aramwit, P. Comparative Clinical Study of Bactigras and Telfa AMD for Skin Graft Donor-Site Dressing. *IJMS* **12**, 5031–5038 (2011).

28. Napavichayanun, S., Ampawong, S., Harnsilpong, T., Angspatt, A. & Aramwit, P. Inflammatory reaction, clinical efficacy, and safety of bacterial cellulose wound dressing containing silk sericin and polyhexamethylene biguanide for wound treatment. *Arch Dermatol Res* **310**, 795–805 (2018).

29. Husmark, J., Morgner, B., Susilo, Y. B. & Wiegand, C. Antimicrobial effects of bacterial binding to a dialkylcarbamoyl chloride-coated wound dressing: an in vitro study. *J Wound Care* **31**, 560–570 (2022).

30. Malmsjö, M., Ingermansson, R., Lindstedt, S. & Gustafsson, L. Comparison of bacteria and fungus-binding mesh, foam and gauze as fillers in negative pressure wound therapy – pressure transduction, wound edge contraction, microvascular blood flow and fluid retention. *International Wound Journal* **10**, 597–605 (2013).

31. Haycocks, S. & Chadwick, P. Use of DACC- coated dressings in diabetic foot ulcers: A case series. **14**, (2011).

32. Nielsen, A. M. & Andriessen, A. Prospective Cohort Study on Surgical Wounds Comparing a Polyhexanide-Containing Biocellulose Dressing with a Dialkyl-Carbamoyl-Chloride–Containing Hydrophobic Dressing. *Advances in Skin & Wound Care* **25**, 409–413 (2012).

33. Bateman, S. D. Evidence is building to support using a DACC-coated antimicrobial wound contact layer with NPWT. *Wounds UK* **11**, 82–86 (2015).

34. Stanirowski, P. J., Bizoń, M., Cendrowski, K. & Sawicki, W. Randomized Controlled Trial Evaluating Dialkylcarbamoyl Chloride Impregnated Dressings for the Prevention of Surgical Site Infections in Adult Women Undergoing Cesarean Section. *Surgical Infections* **17**, 427–435 (2016).

35. Reddersen, K., Tittelbach, J. & Wiegand, C. 3D Biofilm Models Containing Multiple Species for Antimicrobial Testing of Wound Dressings. *Microorganisms* **10**, 2027 (2022).

36. Wiegand, C. *et al.* A standardized wound infection model for antimicrobial testing of wound dressings in vitro. *International Wound Journal* **21**, e14811 (2024).

37. Wiegand, C., Eberlein, T. & Andriessen, A. Antibacterial activity of polihexanide formulations in a co‐culture of HaCaT keratinocytes and *Staphylococcus aureus* and at different pH levels. *Wound Repair Regeneration* **25**, 423–431 (2017).

38. Eberlein, T. *et al.* Comparison of PHMB-containing dressing and silver dressings in patients with critically colonised or locally infected wounds. *J Wound Care* **21**, 12–20 (2012).

39. Lenselink, E. & Andriessen, A. A cohort study on the efficacy of a polyhexanide-containing biocellulose dressing in the treatment of biofilms in wounds. *J Wound Care* **20**, 534–539 (2011).

40. Piatkowski, A., Drummer, N., Andriessen, A., Ulrich, D. & Pallua, N. Randomized controlled single center study comparing a polyhexanide containing bio-cellulose dressing with silver sulfadiazine cream in partial-thickness dermal burns. *Burns* **37**, 800–804 (2011).
